# Purple foliage coloration in tea (*Camellia sinensis* L.) arises from activation of the R2R3-MYB transcription factor CsAN1

**DOI:** 10.1038/srep32534

**Published:** 2016-09-01

**Authors:** Binmei Sun, Zhangsheng Zhu, Panrong Cao, Hao Chen, Changming Chen, Xin Zhou, Yanhui Mao, Jianjun Lei, Yanpin Jiang, Wei Meng, Yingxi Wang, Shaoqun Liu

**Affiliations:** 1 College of Horticulture, South China Agricultural University, Guangzhou 510642, China; 2 College of Forestry and Landscape Architecture, South China Agricultural University, Guangzhou 510642, China

## Abstract

Purple foliage always appears in *Camellia sinensis* families; however, the transcriptional regulation of anthocyanin biosynthesis is unknown. The tea bud sport cultivar ‘Zijuan’ confers an abnormal pattern of anthocyanin accumulation, resulting in a mutant phenotype that has a striking purple color in young foliage and in the stem. In this study, we aimed to unravel the underlying molecular mechanism of anthocyanin biosynthetic regulation in *C. sinensis*. Our results revealed that activation of the R2R3-MYB transcription factor (TF) anthocyanin1 (CsAN1) specifically upregulated the bHLH TF CsGL3 and anthocyanin late biosynthetic genes (LBGs) to confer ectopic accumulation of pigment in purple tea. We found CsAN1 interacts with bHLH TFs (CsGL3 and CsEGL3) and recruits a WD-repeat protein CsTTG1 to form the MYB-bHLH-WDR (MBW) complex that regulates anthocyanin accumulation. We determined that the hypomethylation of a CpG island in the *CsAN1* promoter is associated with the purple phenotype. Furthermore, we demonstrated that low temperature and long illumination induced *CsAN1* promoter demethylation, resulting in upregulated expression to promote anthocyanin accumulation in the foliage. The successful isolation of *CsAN1* provides important information on the regulatory control of anthocyanin biosynthesis in *C. sinensis* and offers a genetic resource for the development of new varieties with enhanced anthocyanin content.

Tea (*Camellia sinensis* L.) is an important global commercial crop, and is primarily consumed as a non-alcoholic beverage made from the processed leaves. The tea beverage yields many health benefits to humans due to the extensive secondary metabolites found in tea leaves, including flavonoids, theanine, and volatile oils^1^. Flavonoids, including catechins, anthocyanins, proanthocyanins, flavonols and phenolic acids, are a large group of phenolic secondary metabolites and have potential beneficial properties for human health^2^.

Anthocyanins are the largest group of water-soluble pigments in the plant kingdom and belong to the family of compounds known as flavonoids. Anthocyanins display a wide range of biological functions, such as attracting pollinators and seed dispersers and protecting plants against various biotic and abiotic stresses^2^,^3^. To date, most anthocyanin biosynthetic pathway genes and numerous regulatory factors have been identified from studies in Arabidopsis, maize, petunia, apple, and other plant species^4^,^5^,^6^,^7^. Transcriptional regulation of structural genes appears to be a major mechanism by which anthocyanin biosynthesis is regulated in plants^2^. In many species studied to date, regulation occurs through a complex of MYB transcription factors (TFs), basic helix-loop-helix (bHLH) TFs, and WD-repeat proteins in the MYB-bHLH-WD40 (MBW) complex^2^,^8^. The MYB member of this complex often appears to be the major determinant of variation in anthocyanin pigmentation^6^. Activation of anthocyanin master regulators, including *AtMYB75* in Arabidopsis, *SlANT1* in tomato, *MdMYB10* and *MdMYB110a* in apple, *BoMYB2* in cauliflower, *McMYB10* in crabapple, and *RsMYB1* in radish, is associated with upregulation of the anthocyanin biosynthetic structural genes and results in anthocyanin accumulation^5^,^7^,^9^,^10^,^11^,^12^,^13^. In contrast, loss of function or repression of *VvMYBA1* in grapevine, *MdMYB10* in apple, *EgVIR* in oil palm, and *PcMYB10* in pear leads to color loss in normal anthocyanin-accumulating tissues^14^,^15^,^16^,^17^.

Although many studies have investigated the mechanisms underlying the control of anthocyanin production in flowers, fruits, and model plants, few studies have focused on tea plants. Because *C. sinensis* is difficult to culture in *vitro*, difficult to transform, and has a large genome^1^, the underlying molecular mechanisms of transcriptional control of anthocyanin accumulation in the tea plant remain unknown. Mutant analyses have facilitated gene discovery and elucidation of the regulatory control of anthocyanin biosynthesis^7^,^18^. The purple foliage cultivar ‘Zijuan’ is a somatic mutant selected from the Yunnan Daye cultivar (*Camellia sinensis* var. assamica (Mast.) Kitamura), in which the anthocyanin content was reported to be approximately three times that found in other Chinese purple tea cultivars^19^. Thus, this mutant provides an excellent opportunity to uncover the regulatory control of anthocyanin biosynthesis in tea plants. To obtain more insight into the high levels of anthocyanin accumulation in ‘Zijuan’, a comparative transcriptome strategy was used, with the aim of revealing the underlying molecular mechanisms. We found that the *CsAN1* homologue of *AtMYB113* in Arabidopsis and anthocyanin LBG expression levels were highly correlated with anthocyanin accumulation. Two candidate bHLH partners and a WD-repeat protein were also isolated. Further analysis revealed that the methylation and demethylation of the CsAN1 promoter may be the factor causing purple foliage, thus providing a mechanism by which anthocyanin transcriptional regulation can be modulated differently in plants.

## Results

### Analysis of anthocyanin content in different *C. sinensis* cultivars

**Figure 1:**
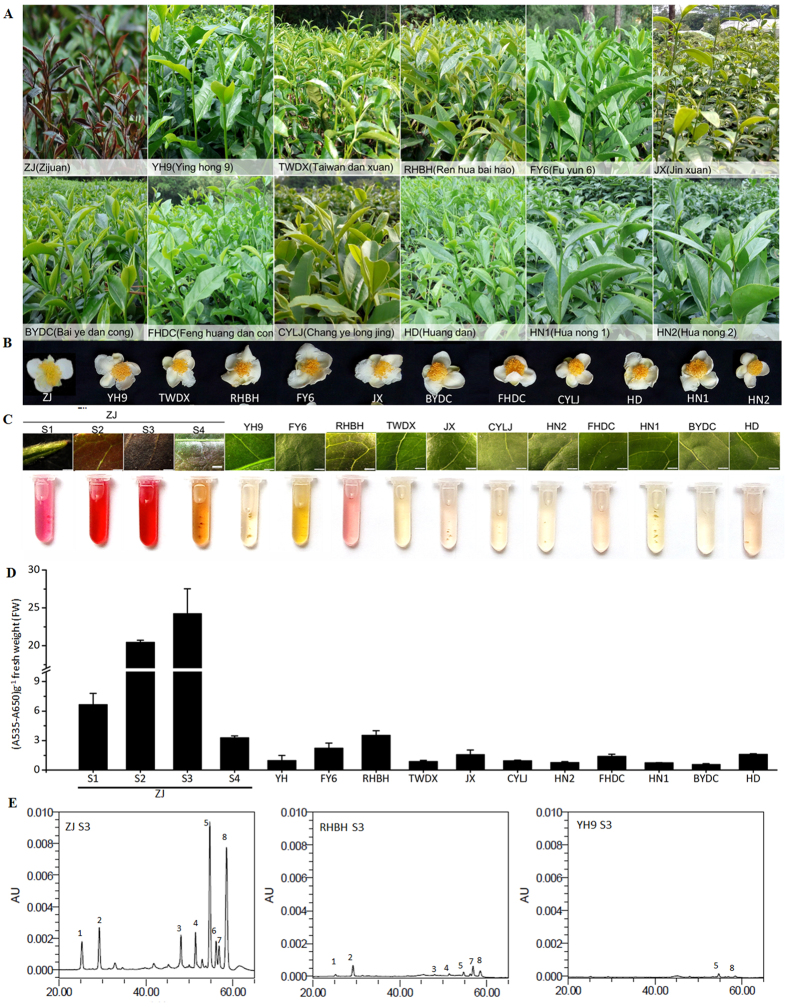
Analysis of anthocyanin content in different *C. sinensis* cultivars. Phenotypes of *C. sinensis* leaves (**A**) and flowers (**B**). (**C**) Close-up of purple foliage cultivar ‘Zijuan’ (‘ZJ’) leaf in stages S1, S2, S3 and S4 and green foliage cultivars leaves in stage S3 and corresponding extraction of anthocyanin. Bar = 1 mm. S1, bud; S2, 7 days after budding (DAB); S3, 15 DAB; S4, 40 DAB. (**D**) Analysis for anthocyanin as shown in (**C**). Error bars on each symbol indicate the mean ± SD of three biological repeats. (**E**) HPLC traces at 520 nm of red and evergreen foliage cultivars ‘ZJ’, ‘RHBH’ and ‘YH9’ in stage S3 leaves. 5, cyanidin-3-*O*-galactoside, 8, delphinidin-3-*O*-galactoside.

The purple foliage tea plants grew and developed normally when compared to green foliage under normal growth conditions in a tea garden. ‘Zijuan’ (‘ZJ’) exhibited a tissue-specific pattern of anthocyanin accumulation. Intense purple coloration was mainly observed in young leaves and stems (Fig. 1A). In contrast, the purple color was almost absent in mature leaves, stems and flower petals (Fig. 1A–C). Consistent with the observed phenotypes, ‘ZJ’ showed an abundant level of anthocyanin accumulation in stage S1 leaves and the highest accumulation in stage S3 leaves, while the stage S4 leaves accumulated little anthocyanin. In contrast, only trace amounts of anthocyanin were detected in stage S3 leaves of green-colored cultivars (Fig. 1D). To examine the composition of anthocyanin accumulated in ‘ZJ’ leaves, we performed HPLC analysis of anthocyanin compound composition. The results suggested that ‘ZJ’ contains two major anthocyanin peaks at 520 nm; cyanidin-3-*O*-galactoside (peak 5) and delphinidin-3-*O*-galactoside (peak 8) were the main coloration anthocyanin components in the ‘ZJ’ leaves (Fig. 1E), which is consistent with previous results in tea plants^20^,^21^. In contrast, anthocyanin peaks were almost undetectable in the ‘RHBH’ and ‘YH9’ cultivars. In general, anthocyanin accumulation in the purple-red foliage of ‘ZJ’ is higher than in other cultivars, indicating that anthocyanin accumulation is responsible for the purple coloration in tea.

### Anthocyanin LBGs and *CsAN1* are upregulated in red foliage

**Figure 2:**
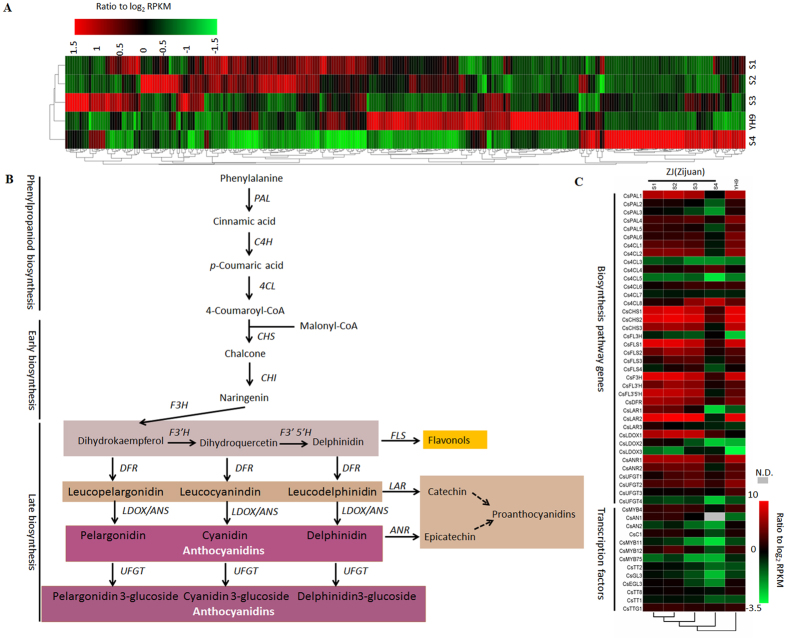
Analysis of transcription data obtained from *C. sinensis* cultivars ‘ZJ’ and ‘YH9’. (**A**) Hierarchical clustering analysis of 308 phenylpropanoid and flavonoid pathway related transcripts unigenes based on RNA-seq profiles data. (**B**) Simplified model of the flavonoid biosynthetic pathway in tea plant. (**C**) Phenylpropanoid and flavonoids pathway with related transcripts present in ‘ZJ’ and ‘YH9’, respectively. N.D., undetected.

Genes involved in three secondary metabolic pathways (flavonoid biosynthesis, anthocyanin biosynthesis, and flavone and flavonol biosynthesis pathways) that are related to foliage pigmentation were analyzed using *C. sinensis* transcript unigenes. By mapping to the KEGG reference pathways, a total of 308 unigenes were assigned to the three pathways (Fig. 2A). We examined the expression levels of anthocyanin structural genes and found no significant difference in early biosynthetic genes (EBGs). However, the LBGs *CsF3*′*H, CsF3*′*5*′*H, CsDFR1, CsDFR2, CsLDOX1, CsLDOX2* and *CsLDOX3* showed 12.7-, 6.49-, 2.3-, 10.7-, 36.4-, 37.9- and 7.9-fold higher levels of expression, respectively, in purple-colored foliage than in the corresponding green-colored foliage (Fig. 2B,C). In addition, *CsLAR1, CsLAR2*, and *CsLAR3*, which encode enzymes for catechin biosynthesis, were highly expressed in red foliage, whereas their transcripts were almost undetectable in green foliage (Fig. 2B,C). Moreover, the expression level of anthocyanin LBGs was significantly upregulated in young ‘ZJ’ tea leaves (Fig. 2C). The result was also validated by real time qRT-PCR analysis (Fig. S1). This result demonstrated that accumulation of anthocyanin in tea foliage is regulated at the transcriptional level.

Flavonoid biosynthesis is regulated by the MYB-bHLH-WDR (MBW) transcriptional complex. In particular, a subgroup of R2R3-MYB characterized by the presence of the bHLH interacting signature ([DE]Lx2[RK]x3Lx6Lx3R) in the R2R3 domain and a C-terminus KPRPR[S/T]F motif that is typical of anthocyanin regulators has been well documented in the literature^22^,^23^. Therefore, we investigated anthocyanin biosynthesis-related MYB, bHLH, and WD40 TFs in the *C. sinensis* transcriptome. Of these unigenes, c45468_g1_i1 and c59852_g1_i1 were designated as *anthocyanin1 (CsAN1*) and *anthocyanin2 (CsAN2*), respectively; they encode TFs homologous to *AtMYB113* from *Arabidopsis,* which is related to anthocyanin biosynthesis. In the stage S2 foliage, only *CsAN1* showed 19.4-fold higher expression in red foliage than in green foliage, while no significant difference was observed in *CsAN2* (Fig. 2C). Moreover, compared with mature leaves, *CsAN1* expression was markedly upregulated in young leaves and was highly correlated with transcript levels of anthocyanin LBGs, which is consistent with the tissue-specific pattern of anthocyanin accumulation in ‘ZJ’ tea plants.

We also investigated anthocyanin biosynthesis-related bHLH TFs and WD-repeat genes in the *C. sinensis* leaf transcriptome. Two homologues of *AtGL3* and *AtEGL3 (CsGL3* and *CsEGL3*) and a homologue of *AtTTG1 (CsTTG1*) were identified. *CsGL3* had 1.7-fold higher expression levels in stage S2 purple foliage than in the corresponding green foliage (Fig. 2C). By contrast, the *CsEGL3* and *CsTTG1* expression levels were not significantly different between red and green foliage (Fig. 2C), which is consistent with our real time qRT-PCR results (Fig. S1).

Taken together, the results suggest that *CsAN1* is the candidate gene controlling levels of anthocyanin accumulation in ‘ZJ’ tea.

### Phylogenetic analysis of the identified *C. sinensis* transcriptional regulators

**Figure 3:**
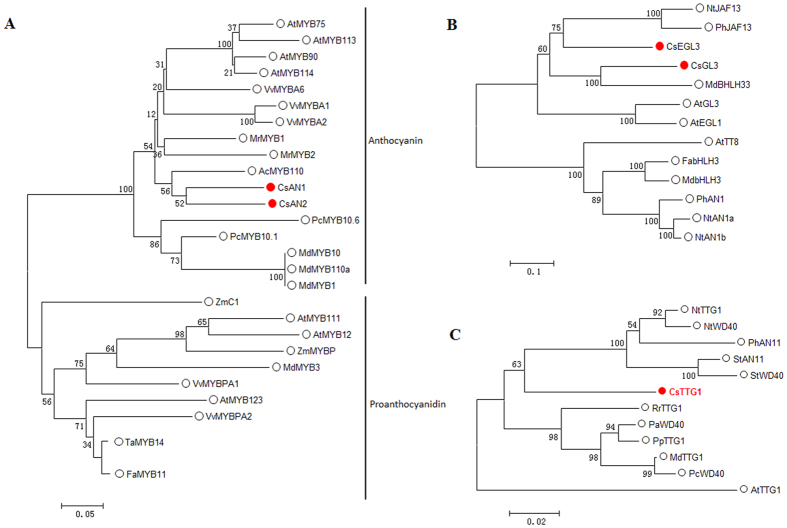
Identification of putative transcriptional regulators involved in anthocyanin biosynthesis in tea. Schematic representation of the phylogenetic relationships existing between the tea MYB (**A**), basic helix–loop–helix (bHLH) (**B**) and TTG1-like (**C**) proteins isolated and used in this study with their closest homologues. Sequences were aligned using Clustal X and phylogeny reconstruction was done using the neighbour-joining method and tested using the bootstrap method with 1000 replicates. Both alignment and phylogenetic analysis were performed using MEGA version 7.

Phylogenetic analysis of the deduced amino acid sequences using the neighbor-joining method was employed to determine the relationships that may exist between the identified *C. sinensis* MYB, bHLH and WD40-repeat regulatory proteins and known TF genes. Phylogenetic analysis indicated that *CsAN1* and *CsAN2* are most similar to the *Actinidia chinensis* anthocyanin regulatory TF *AcMYB110* and share 58% and 56% identity, respectively. Sequence alignment indicated that *CsAN1* and *CsAN2* have a bHLH interacting signature (Fig. S2). The *CsGL3* amino acid sequence is very similar to *MdbHLH33* from apple (61% identity), while the *CsEGL3* amino acid sequence shares 52.6% identity with *Nicotiana tabacum NtJAF13* (Fig. 3B; Fig. S3). Finally, CsTTG1 displays very high similarity to its homologues from *Nicotiana tabacum, NtTTG1*, and *Petunia hybrid, PhAN11* (Fig. 3C; Fig. S4).

### Sub-cellular localization of the CsAN1, CsGL3, CsEGL3, CsTTG1 proteins and transcriptional activity analysis of CsAN1

**Figure 4:**
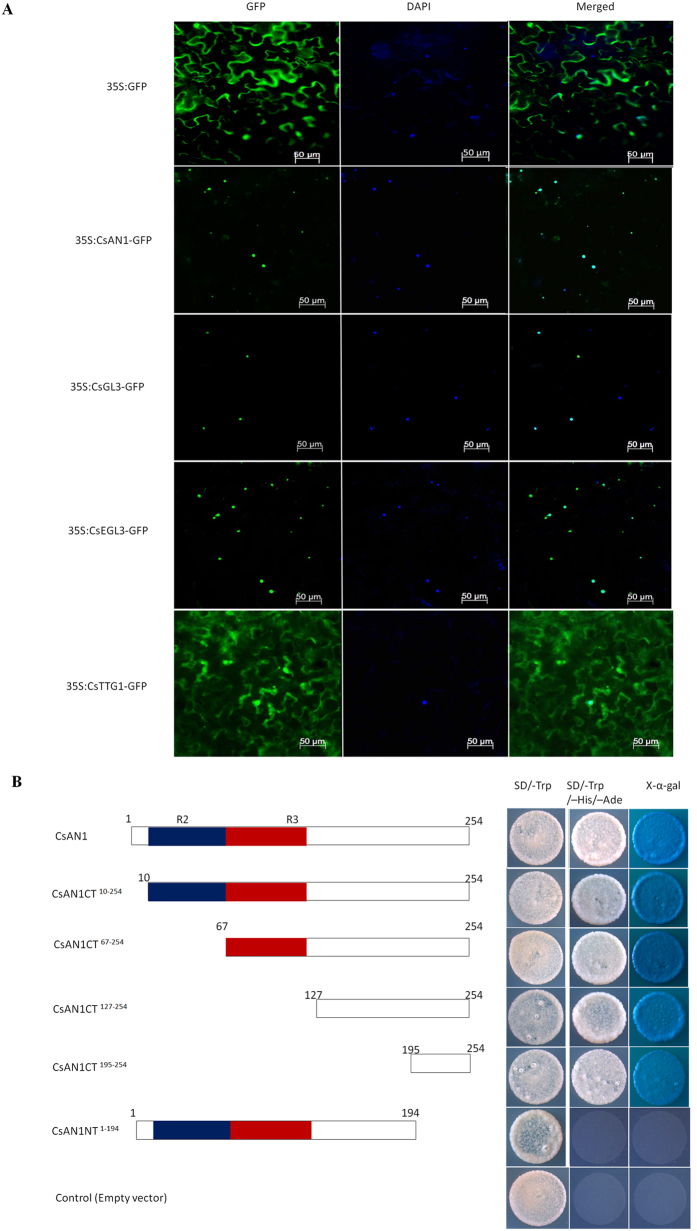
Subcellular localizations of CsAN1, CsGL3, CsEGL3 and CsTTG1 and transactivation assay of CsAN1. (**A**) Subcellular localization of CsAN1, CsGL3, CsEGL3 and CsTTG1 in epidermal cells of *N. benthamiana* leaves. GFP fluorescence was detected 50 hours after infiltration. The nuclei were indicated by DAPI staining. (**B**) Transactivation assay of CsAN1. Either the full length or a truncated ORF of *CsAN1* was fused with pGBKT7, and transformed yeasts were selected on SD/-Trp or SD/-Trp/-His/-Ade/X-α-gal media for 3 d at 30 °C. Transcription activation was monitored by the detection of yeast growth and an α-galactosidase assay.

To determine the subcellular localization of the TFs identified in this work, the full-length coding sequences of each respective TF were fused in frame with the GFP gene. These constructs were transformed into *Agrobacterium* strain GV3101 and then infiltrated in *N. benthamiana* leaves; the fluorescence signals were examined in epidermal cells. As shown in Fig. 4, 35S:GFP proteins were localized in both the cytoplasm and nucleus, whereas the 35S:CsAN1-GFP, 35S:CsGL3-GFP and 35S:CsEGL3-GFP fusion proteins were exclusively localized in the nucleus (Fig. 4A). Interestingly, the fluorescence of 35S:CsTTG1-GFP was not targeted exclusively to the nucleus, and the GFP signal was found in the cytoplasm and cell membrane (Fig. 4A).

The R2R3-MYB TF *CsAN1* is significantly upregulated in purple foliage and localized to the nucleus, implying that it may act as a transcription activator. A transactivation assay indicated that only transformants of pGBKT7 that were fused with the full-length *CsAN1* ORF (covering 1–254 aa) or truncated *CsAN1* ORF fragments expressing C-terminal amino acid sequences (covering 10–254 aa, 67–254 aa, 127–254 aa and 195–254 aa, respectively) grew well in SD/-Trp/-His/-Ade and showed α-galactosidase activity. Truncated *CsAN1* ORF fragments expressing N-terminal amino acid sequences (covering 1–194 aa) or pGBKT7 (negative control) showed no α-galactosidase activity (Fig. 4B). These assays indicated that CsAN1 has transactivation activity, which is attributed to the C-terminal 195–254 residues.

### CsAN1 associates with the *CsLDOX1* and *CsLDOX2* promoters

MYB TFs specially bind to the so-called MYB binding site (MBS). Sequence analysis identified MBS elements in the promoters of *CsLDOX1* and *CsLDOX2* (Fig. S5A), suggesting that they might be the direct targets of CsAN1. Y1H assays were performed to test the association of CsAN1 with the *CsLDOX1* and *CsLDOX2* promoters. The results showed that CsAN1 binds to the promoters of *CsLDOX1* and *CsLDOX2* (Fig. S5B), supporting the hypothesis that CsAN1 is the TF regulating the expression of CsLDOX1 and CsLDOX2.

### CsGL3 and CsEGL3 modulate the intracellular localization of CsTTG1

CsTTG1-GFP was typically found in the cytoplasm and nucleus of epidermal cells (Fig. 4A; Fig. S6D). This intracellular localization appears to be altered in tobacco plants overexpressing CsGL3 and CsEGL3. However, cells co-expressing 35S:CsGL3 or 35S:CsEGL3 with CsTTG1-GFP accumulated GFP mainly in the nucleus (Fig. S6E,F). As a negative control, GFP distribution in wild type, CsGL3-overexpressing and CsEGL3-overexpressing lines appears to be throughout *N. benthamiana* epidermal cells (Fig. S6A–C). Taken together, we hypothesized that the intracellular localization of CsTTG1 depends partly on the bHLH proteins CsGL3 and CsEGL3 in a partially redundant manner. This result revealed that two bHLH TFs may recruit CsTTG1 to form the MYB-bHLH-WDR (MBW) complex that synergistically controls expression.

### CsAN1 and CsTTG1 interact with CsGL3 or CsEGL3 to form a MYB/bHLH/WD-repeat (MBW) protein complex

**Figure 5:**
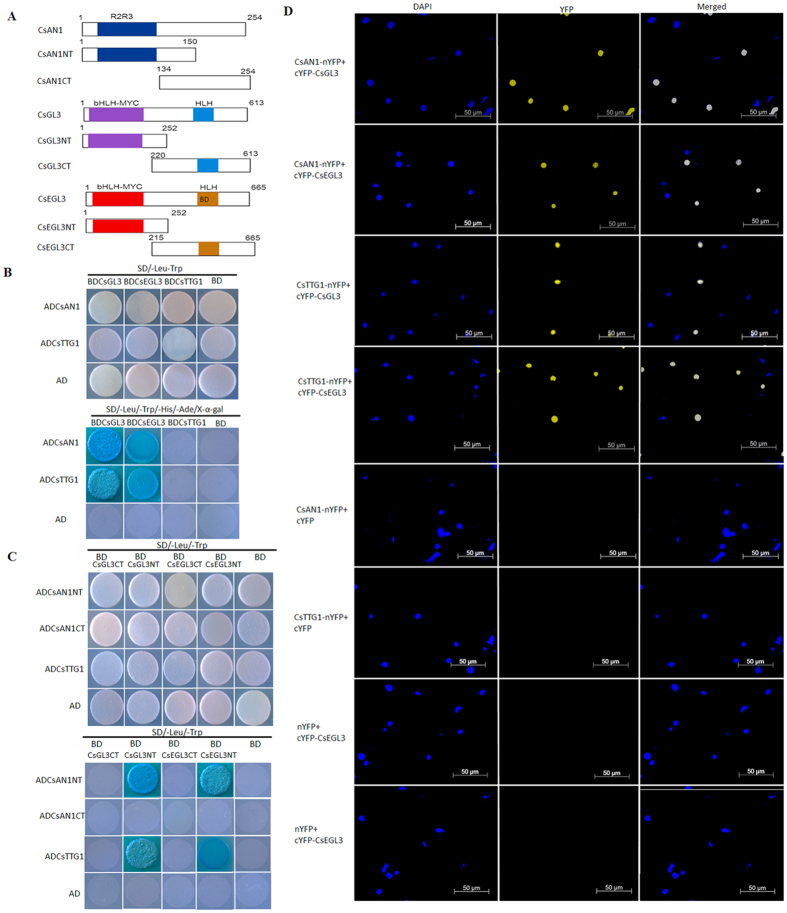
CsAN1 and CsTTG1 interact with CsGL3 and CsEGL3. (**A**) Schematic diagram of CsAN1, CsGL3 and CsEGL3 domain constructs. (**B**) Y2H assays show that CsAN1 and CsTTG1 interact with CsGL3 and CsEGL3 in yeast. (**C**) Y2H assays show that CsAN1NT and CsTTG1 interact with CsGL3NT and CsEGL3NT in yeast. (**D**) BiFC assay to detect the interactions of CsAN1 and CsTTG1 (fused with N-terminal fragment of YFP) with CsEGL3, CsGL3 (fused with C-terminal fragment of YFP). Construct pairs indicated on the left were co-expressed in leaves of *N. benthamiana* and and visualized using confocal microscopy. Expressions of CsAN1, CsGL3, CsEGL3, CsEGL3 or CsTTG1 alone were used as negative controls. YFP fluorescence was detected 50 h after infiltration. The nuclei are indicated by DAPI staining.

To investigate whether CsAN1, CsGL3, CsEGL3 and CsTTG1 form a MBW complex to regulate anthocyanin accumulation Y2H was used (Fig. 5A). We found that CsAN1 interacts with CsGL3 and CsEGL3, but not with CsTTG1 (Fig. 5B). Similarly, CsTTG1 interacts with CsGL3 and CsEGL3 (Fig. 5B).

We examined which domains of CsGL3 and CsEGL3 in the MYB/bHLH/WD-repeat complex are responsible for interacting with the CsAN1 and CsTTG1 proteins. The bHLH members (CsGL3 and CsEGL3) were divided into N-terminal fragments (CsGL3NT and CsEGL3NT) containing a bHLH-MYC binding domain and C-terminal fragments (CsGL3CT and CsEGL3CT) containing the bHLH DNA binding domain (Fig. 5A). CsAN1 was similarly divided into N-terminal fragments containing the R2R3 DNA binding domain and C-terminal fragments to produce CsAN1NT and CsAN1CT (Fig. 5A). As shown in Fig. 5C, the bHLH member N-terminal fragments (CsGL3NT and CsEGL3NT), but not the C-terminal fragments (CsGL3CT and CsEGL3CT), of these transcription factors exhibited interactions with CsAN1NT and CsTTG1 in yeast. These results suggest that the N-terminal domains of CsGL3 and CsEGL3 are responsible for the interaction with CsAN1 and CsTTG1.

We next adopted BiFC assays^24^ to verify the interactions of CsAN1 and CsTTG1 proteins with CsGL3 and CsEGL3 in planta. The N-terminal fragment of yellow fluorescent protein (nYFP) was ligated with CsAN1 and CsTTG1 to produce CsAN1-nYFP and CsTTG1-nYFP. CsGL3 and CsEGL3 were individually fused with the C-terminal fragment of YFP (cYFP). CsAN1-nYFP and CsTTG1-nYFP were transiently co-expressed with cYFP-CsGL3 or cYFP-CsEGL3 in *N. benthamiana* leaves. We found that co-expression of CsAN1-nYFP with cYFP-CsGL3 or cYFP-CsEGL3 resulted in strong YFP fluorescence in the nuclei of epidermal cells in *N. benthamiana* leaves (Fig. 5D). Similar results were observed for co-expression of CsTTG1-nYFP with cYFP-CsGL3 or cYFP-CsEGL3 (Fig. 5D). In contrast, co-expression with the negative control combinations failed to generate YFP fluorescence.

Collectively, these results implied that CsAN1 and CsTTG1 proteins physically interact with the bHLH to form a MYB/bHLH/WD-repeat complex.

### *CsLDOX2* promoter activity is regulated by MBW protein complexes

To predict the transcriptional regulatory roles of MBW protein complexes, a dual luciferase assay with the *CsLDOX2* promoter was used in this study. Based on the results, compared to the empty vector, CsAN1 induced an approximately 4.2-fold increase in *CsLDOX2* promoter activity (Fig. S7A). When co-expressed with CsAN1, CsGL3 or CsEGL3 showed a significant synergistic effect, and *CsLDOX2* promoter activity was stimulated over 25.6- and 13.2-fold, respectively (Fig. S7B). Moreover, the highest activity, an approximately 40-fold increase, was observed when CsAN1, CsTTG1 and CsGL3 were transformed simultaneously. emphasizing the fact that these three proteins act together to activate their target promoters.

### Altered MBW complex gene expression changes anthocyanin accumulation in plants

**Figure 6:**
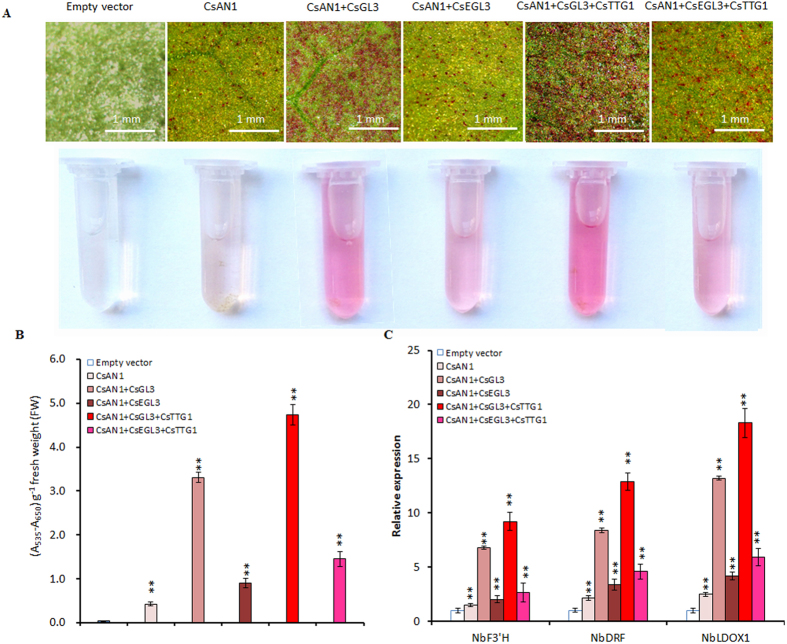
Transient overexpression of *C. sinensis* MBW complex genes induced anthocyanin biosynthesis in *N. benthamiana* leaves. (**A**) *N. benthamiana* leaves in 10 days after transformation with: Empty vector, *CsAN1, CsAN1* + *CsGL3, CsAN1* + *CsEGL3, CsAN1* + *CsGL3* + *CsTTG1* and *CsAN1* + *CsEGL3* + *CsTTG1*, respectively. (**B**) Anthocyanin contents as shown in (**A**). (**C**) Expression levels of *NbF3*′*H* (AB289449), *NbDFR* (AB289448) and *NbLDOX1* (AB723683) in *N. benthamiana* leaves transformed with indicated vectors. Error bars are the SD for five replicate reactions. Asterisks indicate significant differences (p < 0.01) by Student’s t-test.

For MBW-induced anthocyanin pigment accumulation in *N. benthamiana, CsAN1, CsAN1*/*CsGL3, CsAN1*/*CsEGL3, CsAN1*/*CsGL3*/*CsTTG1* and *CsAN1*/*CsEGL3*/*CsTTG1* were syringe-infiltrated into the underside of expanding tobacco leaves. Pigmentation was evident at the infiltration points as early as 4 days post-infiltration for *CsAN1*/*CsGL3*/*CsTTG1*. The degree of pigmentation gradually increased over the experimental period of up to 10 days (Fig. 6A). However, no coloring was observed at the infiltration sites 10 days after transformation with empty vector (Fig. 6A). Consistent with the visual assessment of red pigmentation, leaves collected at 10 days post-infiltration had undetectable levels of anthocyanin in leaf disks infiltrated with empty vector and different levels in leaf disks infiltrated with *CsAN1* and *CsGL3* or *CsEGL3, CsAN1* and *CsTTG1, CsGL3* or *CsEGL3* (Fig. 6A,B).

To elucidate the molecular mechanism behind this effect of *C. sinensis* MBW complex on anthocyanin biosynthesis in tobacco, a transcript accumulation study of MBW genes and anthocyanin LBGs was performed using quantitative RT-PCR (qRT-PCR). The results revealed that the expression levels of MBW-related genes were not significantly different when infiltrated alone or co-infiltrated with its partners, whereas LBG transcript accumulation was slightly increased when infiltrated with *CsAN1* alone compared with the empty vector (Fig. 6C; Fig. S8). Co-expression of *CsAN1*/*CsGL3* or *CsAN1*/*CsGL3*/CsTTG1 resulted in higher transcript levels of anthocyanin LBGs than in the corresponding leaf disks infiltrated with *CsAN1*/*CsEGL3* or *CsAN1*/*CsEGL3*/CsTTG1, consistent with the measured anthocyanin content (Fig. 6C).

### Hypomethylation of the *CsAN1* promoter resulted in higher expression in purple-colored foliage

To investigate the molecular basis of the higher expression levels of *CsAN1* in the purple foliage of tea, the promoter of *CsAN1* was isolated from ‘ZJ’, ‘RHBH’, ‘FY6’ and ‘YH9’. We sequenced the PCR products of each of these fragments from three independent reactions in the four cultivars. The promoters contained a minisatellite polymorphism (SSR) and showed limited variation between the cultivars (Fig. S9A). Interestingly, further analysis indicated that these promoters contained Gypsy-18 and SZ-67 elements (Fig. S9B), both of which are class I long terminal repeat retrotransposons. These elements may attract epigenetic changes to activate or deactivate adjacent host genes.

**Figure 7:**
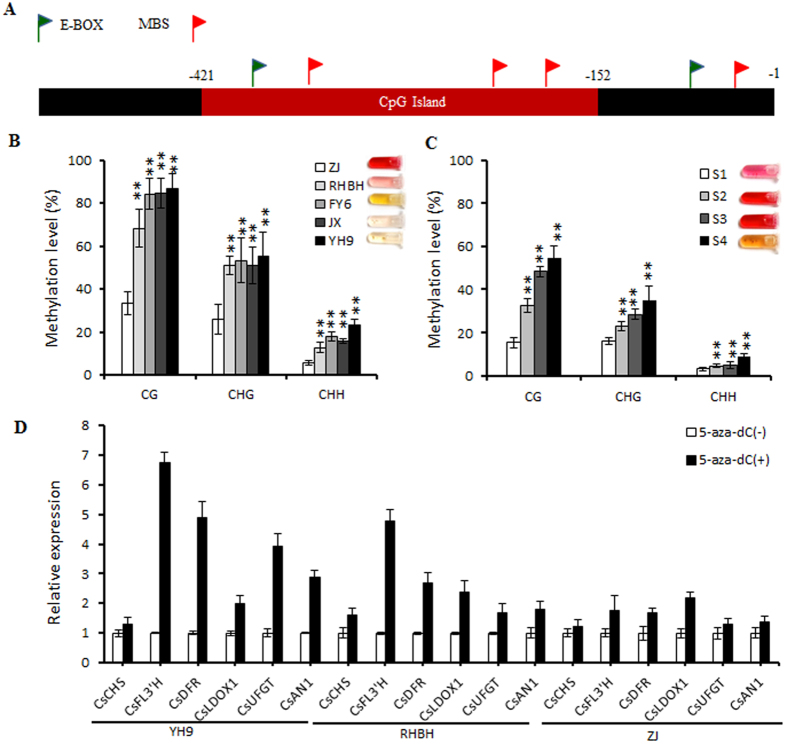
DNA methylation analysis of the *CsAN1* promoter. (**A**) Schematic representation of CsAN1 promoter. Red box indicate CpG island. (**B**) DNA methylation status of bisulfite-sequenced CpG island in five tea cultivars and (**C**) ‘ZJ’ leaf at four developmental stages. CG, CHG and CHH refer to the three different contexts of cytosines, in which H represents nucleotide A, C or T. Each data point represents a mean ± SD of three independent *g*DNA extractions with three independent technical replicates. Five independent clones from each reaction were sequenced and analyzed. (**D**) Real-time reverse transcription-PCR analyses of CsAN1 and anthocyanin biosynthesis related genes expression in ‘YH9’, ‘RHBH’ and ‘ZJ’ leaves treated with (+) or without (−) 5-aza-dC, an inhibitor of DNA methylation. Tea *Actin* was used as a control. Values are means ± SD of three biological replicates. Asterisks indicate significant differences (p < 0.01) by Student’s t-test.

To verify this hypothesis, we analyzed the methylation levels of cytosine in the *CsAN1* promoter of green and purple foliage tea cultivars by bisulfite sequencing (BSP)-PCR. The methylation levels of the CG, CHG, and CHH cytosines (where H is A, C or T) in the CpG island of the *CsAN1* promoter (5′ upstream of start codon -152 to -421) were significantly decreased in the high anthocyanin cultivars compared to the low anthocyanin cultivars (Fig. 7B). Moreover, to examine whether there is alteration in the dynamics of methylation in the *CsAN1* promoter during leaf development, the methylation activity of the CpG island was assessed in the ‘ZJ’ leaf during the four stages using the bisulfite sequencing approach. The results demonstrated that ‘ZJ’ exhibited markedly different methylation levels during leaf development. The methylation levels of cytosine progressively increased with leaf maturation, resulting in a high methylation level in stage S4 leaves (Fig. 7C).

To further confirm whether the ectopic expression of *CsAN1* results in hypomethylation of the promoter region, we treated the tea plants with 5-aza-2′ deoxycytidine (5-aza-dC), an inhibitor of DNA methylation^25^. Treatment with 5-aza-dC increased the expression of *CsAN1* 2.88-fold in ‘YH9’, 1.79-fold in ‘RHBH’ and 1.39-fold in ‘ZJ’, leading to activation of anthocyanin LBG expression (Fig. 7D). These results indicate that DNA methylation in the 5′ upstream sequence of *CsAN1* plays an essential role in the regulation of *CsAN1* expression.

### Temperature and light alter the expression of the MBW complex and anthocyanin biosynthetic genes

**Figure 8:**
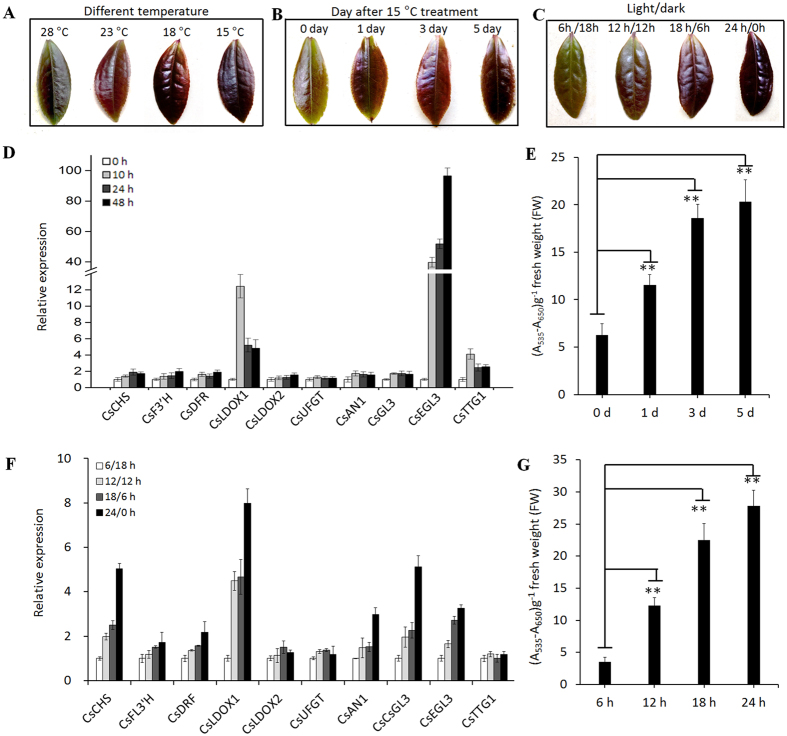
Different environmental conditions alter anthocyanin accumulation by changing the expression of anthocyanin related MBW-complex and biosynthetic genes. (**A**) Leaves harvested from the different temperature conditions. (**B**) Leaves harvested in 1, 3, 5 day after 15 °C treatment plants. (**C**) The ‘Zijuan’ tea leaves harvested from different photoperiod conditions. (**D**) The genes expression profile after 15 °C treatment with 0, 10, 24, 48 h. (**E**) Low temperature (15 °C) increases the accumulation of anthocyanin in leaves. The 0, 1, 3, 5 day indicates anthocyanin levels after 15 °C treatment. (**F**) The genes expression profile under different photoperiod conditions. (**G**) The anthocyanin levels under different photoperiod conditions. Error bars on each symbol indicate the mean ± SD of three replicate reactions. Asterisks indicate significant difference (P < 0.01) between combinations by Student’s t-test.

Under a high temperature of 28 °C, ZJ tea leaves showed only slight red pigmentation indicating regulation of anthocyanin biosynthesis is temperature sensitive. To understand the effect of temperature on coloration and the expression of anthocyanin-related genes in leaves, three-year-old ‘ZJ’ trees were placed at 28 °C, 23 °C, 18 °C, and 15 °C to test the effects of low temperatures. Compared with a high temperature of 28 °C, low temperatures dramatically promoted red coloration in ‘ZJ’ tea leaves (Fig. 8A). In the high temperature treatment of 28 °C, we reset the temperatures as low as 15 °C, without changing the other conditions. The tree leaves showed a significant accumulation of anthocyanin content (Fig. 8B,E). Correspondingly, the gene transcripts of both the anthocyanin biosynthetic genes and the transcriptional activation complex increased (Fig. 8D).

To understand the effect of light on coloration and the expression of anthocyanin biosynthesis-related genes in leaves, three-year-old ‘ZJ’ trees were used to conduct 6-h, 12-h, 18-h and 24-h light period lighting treatments. The leaves displayed marked red pigmentation after 15 days of long light, whereas the 6-h light period leaves remained slightly red colored (Fig. 8C). Accordingly, in comparison with short light, *CsAN1, CsGL3* and *CsEGL3* expression increased in the long light conditions, and the anthocyanin biosynthetic gene transcripts were increased (Fig. 8F). Thus, longer light periods enhanced anthocyanin biosynthesis and accumulation in leaves (Fig. 8G).

These results indicated that low temperatures and long illumination times promote anthocyanin accumulation due to partial or complete activation of *C. sinensis* anthocyanin biosynthesis-related MBW gene expression.

### Low temperature and a long photoperiod induce *CsAN1* promoter demethylation

The transcription of CsAN1 appears to be elevated in response to environmental factors. To examine whether DNA methylation in the *CsAN1* promoter is plastic under different environmental factors, the methylation levels of ‘ZJ’ stage S2 leaves were evaluated using the bisulfite sequencing approach. The results demonstrated that ‘ZJ’ exhibited markedly different methylation levels under four different temperature treatments. The methylation levels of cytosine progressively increased with temperature increases, resulting in a high methylation level under 28 °C treatment (Fig. S10A). Moreover, we detected that CsAN1 promoter methylation was altered under different illumination conditions. A high methylation level of the CsAN1 promoter was observed under short light, whereas it was notably decreased when the illumination time was increased (Fig. S10B). Our results indicated that environmental factors regulate ‘ZJ’ tea anthocyanin biosynthesis, which partially occurs through *CsAN1* promoter methylation and demethylation.

## Discussion

**Figure 9:**
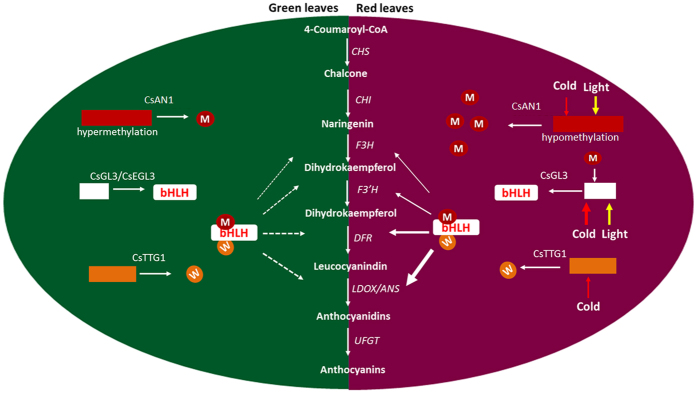
Model showing the molecular mechanism of the purple-coloration in *Camellia sinensis* foliage. The hypomethylation of the *CsAN1* promoter activated the expression of *CsAN1*. CsAN1 interact with CsGL3 to recruits CsTTG1 to form MBW protein complexe, subsequently activation the expression of anthocyanin LBGs. Environment factors, low temperature and long-photoperiod activation expression of MBW protein complex, which significantly activating on the anthocyanin pathway. As a result, the biosynthesis of anthocyanin is greatly increased, causing the purple-coloration.

We found that red foliage is associated with high anthocyanin accumulation (Fig. 1), which is consistent with increased transcript levels of anthocyanin LBGs, including *CsF3*′*H, CsF3*′5′*H, CsDFRs* and *CsLDOXs*. This result indicates that a high accumulation of anthocyanin in ‘ZJ’ foliage may be attributed to up-regulation of the R2R3-MYB and/or bHLH genes. The activation of R2R3-MYB TFs leads to the up-regulation of regulators and/or anthocyanin biosynthetic genes that are associated with red pigmentation in many crops, such as apple, peach, pear, purple cauliflower, tomato, plum and crabapple^3^,^5^,^7^,^10^,^13^. In this study, we identified two anthocyanin biosynthesis-related R2R3-MYB TFs, but only the *CsAN1* homolog of *AcMYB110* from *Actinidia*^26^ was strongly upregulated in young foliage of the ‘ZJ’ cultivar, indicating that it is a master anthocyanin regulator in ‘ZJ’ tea plants (Fig. 2C; Fig. S1). Interestingly, unlike *AcMYB110* control of kiwifruit petal colour pigmentation, the activation of *CsAN1* in ‘ZJ’ led to anthocyanin-specific accumulation in young leaves and stems but not in flowers, indicating that its function varies in different species. We also detected evidence of increased transcript levels of *CsGL3*, suggesting that, in tea, *CsGL3* is positively regulated by *CsAN1* (Fig. 2; Fig. S1). Although the expression of *MdMYB10* does not increase the expression of a bHLH TF in apple^5^, *AtMYB75* regulates the expression of *AtTT8* in Arabidopsis^27^, while its homologous gene *BoMYB2* appears to strongly upregulate its bHLH interactor *BobHLH1* in cauliflower^7^. Similarly, *AN2* and *AN4*, two genes encoding MYB transcription factors, activate the *AN1* bHLH TF in petunia^28^. Our results demonstrate that *CsAN1* and *CsGL3* likely function together to coordinately regulate several transcripts of anthocyanin LBGs to confer anthocyanin accumulation in the purple foliage of ‘ZJ’ tea (Fig. 9).

The activity of MYB-like genes has been suggested to be the primary cause of natural variation in anthocyanin pigmentation in plants^16^. The gain or loss of function of the master anthocyanin regulator *MYB* always disrupts anthocyanin accumulation, and these changes can be caused by sequence mutations in the *MYB* coding or promoter regions^16^,^17^,^18^,^29^ or by DNA methylation or demethylation in the promoter^15^,^17^,^30^. In this study, the *CsAN1* promoter showed limited variation between varieties, but this variation cannot explain the phenotype between green foliage and red foliage in tea plants. However, a methylation analysis of the *CsAN1* promoter revealed that the methylation level of the CpG island was inversely correlated with the *CsAN1* expression level and anthocyanin content, indicating that a low methylation level of the *CsAN1* promoter in ‘ZJ’ may confer intense anthocyanin accumulation (Fig. 7). Similar hypermethylation in the *MYB* promoter of the anthocyanin regulators *MzP1* in *Zea mays, PcMYB10* in *Pyrus*, and *MdMYB10* in *Malus domestica* are also associated with low anthocyanin content^15^,^17^,^30^,^31^. While other studies have investigated MYB hypermethylation^15^,^17^,^30^, the relationship of MYB methylation in the CsAN1 clade to the regulation of anthocyanin biosynthesis in vegetative tissues has not been reported until this study (Fig. 9).

Interestingly, the CsAN1 promoter microsatellite contained many G-rich SP1-like elements that inhibit CpG methylation^32^,^33^. The Gypsy LTR retrotransposon element in the *CsAN1* promoter may attract epigenetic changes to regulate neighboring gene expression. In Arabidopsis, regulation of DNA methylation of transposable elements and tandem repeats contributes to the regulation of adjacent host gene expression^34^,^35^. In this study, promoter retrotransposon methylation and demethylation may associate with CsAN1 expression, with the mechanism underlying this being of interest for further work.

The biosynthesis of pigments in many plants is affected by environmental conditions^36^,^37^. Numerous studies have shown that light, temperature and phytohormones can affect the expression of anthocyanin regulators and structural genes^23^,^27^,^38^,^39^. In apples, high temperature decreases anthocyanin content and down-regulates flavonoid biosynthesis and *MdMYB10* gene expression^23^. In contrast, low environmental temperatures promote anthocyanin accumulation in apple by up-regulating the expression of *MdbHLH3* in the *MdMYB1* transcriptional complex^40^. Light significantly increases the accumulation of flavonoids, and the expression of their biosynthetic genes has been partly elucidated^38^. Arabidopsis *AtMYB75,* Arabidopsis *AtMYB90* and its homologous gene *MdMYB1* in apple are crucial regulators of light-induced anthocyanin biosynthesis, and their degradation requires the CONSTITUTIVELY PHOTOMORPHOGENIC1 (COP1) ubiquitin-dependent pathway in the dark^41^,^42^. Similarly, several recent reports have described jasmonate-mediated anthocyanin accumulation through upstream control of MBW complex activity^39^,^43^. However, these studies do not fully explain the underlying mechanism of up-regulation and down-regulation of anthocyanin regulators in specific environmental conditions. It was reported that cold-induced expression of maize *ZmMI1* results in severe demethylation in core promoter regions^44^. In this study, we found that the promoter DNA methylation levels of the anthocyanin master regulator *CsAN1* were highly plastic to different environmental factors. Interestingly, the *CsAN1* promoter comprises numerous *cis*-acting elements, including MYB-, MYC-recognizing sites, light-responsive elements, and phytohormone-responsive elements within the retrotransposon, which may potentially be involved in regulating the *CsAN1* response to different environmental circumstances. This result suggested that methylation and demethylation within the *cis*-acting elements may not only regulate the binding of relevant transcription factors but also affect the environmental response (Fig. S10).

In addition to *CsAN1*, we found that low temperatures increased the expression of *CsGL3, CsEGL3* and *CsTTG1*, while long light periods increased the transcription of *CsGL3* and *CsEGL3* but not *CsTTG1*. Under both low temperatures and long illumination periods, the transcription of *CsLDOX1* was markedly elevated. The link between environmental conditions affecting anthocyanin accumulation and the transcription levels of the corresponding genes indicated that the anthocyanin-related MBW genes and *CsLDOX1* are pivotal in the anthocyanin pathway.

## Materials and Methods

### Anthocyanin measurement

Three-year-old *C. sinensis* plant leaves were obtained from the tea garden of South China Agricultural University and used for the analysis of anthocyanin content. Anthocyanin was measured as described previously^40^. The pre-weighed sample leaves were placed into a 1 ml extraction buffer (18% 1-propanol, 1% HCl, and 81% water), boiled for 3 minutes and then incubated in darkness overnight at room temperature. Two absorbencies (A535 and A650) of the extracts were measured spectrophotometrically. The amount of anthocyanin was reported as (A535–A650) g^−1^ fresh weight (FW).

### HPLC analysis

The indicated samples (approximately 0.5 g fresh weight) as shown in the results were extracted with 5 mL of methanol/water/acetic acid (85: 15: 1) extract solution in a test tube at 4 °C in the dark for 72 h, with shaking every 6 h. The anthocyanin standards (Sigma-Aldrich) or tea samples were injected into an XSelect HSS C-18 SB column (4.6 × 250 mm, 5 μm, Waters Technologies) and separated using 5% formic acid (A) and 100% methanol (B) as mobile phases on a Waters Alliance series HPLC system. Detection was performed at 520 nm for anthocyanin^45^.

### RNA-seq, *de novo* assembly, and sequence analysis

Total RNA was extracted at four different stages from ‘ZJ’: buds (S1), 7 DBA (S2), 15 DBA (S3), and 40 DBA (S4); total RNA was extracted from the foliage at S2 for ‘YH9’ (the Yunnan Daye cultivar), which was the same as that used for measuring the anthocyanin content. The cDNA library was sequenced on the Illumina HiSeq2000 platform, and the results were analyzed by the Annoroad Gene Technology Corporation (Beijing, China).

### Isolation of genes involved in anthocyanin biosynthesis and phylogenetic analysis

For identifying anthocyanin biosynthetic structural genes and regulators from the transcriptome database, we began with simple keyword searches and confirmed each search result with BLAST searches. The ORFs of the anthocyanin biosynthetic regulators were cloned by PCR. A phylogenetic tree was constructed using the Molecular Evolutionary Genetics Analysis (MEGA) software version 7 through the neighbor-joining method, and sequence alignment was performed using the CLUSTAL X program. The gene accession IDs are: CsAN1 (KU745295), CsAN2 (1896250) PcMYB10.1 (AKV89247), PcMYB10.6 (KP772286.1), AcMYB110a (AHY00342), MdMYB1 (ADQ27443.1), MdMYB3 (AEX08668), MdMYB10 (ACQ45201.1), MdMYB110a (BAM84362.1), AtMYB90/PAP2 (75338996), AtMYB75/PAP1 (75333682), AtMYB123/TT2 (27151707), AtMYB113 (Q9FNV9), AtMYB114 (Q9FNV8.1), VvMYBA1 (BAD18977), PcMYB10.6 (AKV89252.1), PcMYB10.1 (AKV89247.1), MrMYB1 (ADG21957), MrMYB2 (ADG21958), TaMYB14 (AFJ53059), FaMYB11 (AFL02461), VvMYBPA1 (NP_001268160), VvMYBPA2 (ACK56131), ZmMYBP (NP_001278607), ZmC1 (1613412E), NtJAF13 (AHY00341), PhJAF13 (AAC39455.1), AtGL3 (75309232), AtEGL3 (34222624), MdbHLH33 (ABB84474.1), MdbHLH3 (ADL36597), PhAN1 (AAG25928.1), NtAN1a (AEE99257.1), NtAN1(AEE99258.1), AtTT8 (Q9FT81.2), FabHLH3 (AFL02463.1), AtTTG1 (CAB45372.1), MdTTG1 (ADI58760.1), NtTTG1 (ACJ06978.1), NtWD40 (AIU39032.1), PhAN11 (AAC18914.1), StAN11 (AEF01097.1), StWD40 (AGC31678.1), and FaTTG1 (AFL02466.1).

### Real time qPCR analysis

Total RNA was extracted from tissues as indicated in the figures, and 1 μg of RNA from each sample was used for the reverse transcription reaction using a PrimeScript^TM^ RT reagent kit with gDNA eraser (Takara, Japan). Quantitative real-time PCR analysis was performed on a LightCycler 480 Real-Time PCR System according to the manufacturer’s instructions (Roche); the qPCR program was performed as described previously^46^. As an internal control, the *Actin* transcript was used to quantify the relative transcript levels of each target gene in each sample. The values represent the mean of three biological replicates.

### Subcellular localization

The coding sequences of *CsAN1, CsGL3, CsEGL3* and *CsTTG1* were cloned into a pEGFP vector for fusion with enhanced GFP under control of the 35S promoter to generate the 35S: CsAN1-GFP, 35S:CsGL3-GFP, 35S:CsEGL3-GFP and 35S:CsTTG1-GFP constructs, respectively. *Agrobacterium* containing the indicated constructs was resuspended in infiltration buffer (0.2 mM acetosyringone, 10 mM MgCl_2_, and 10 mM MES, and infiltrated into *N. benthamiana* leaves using a needleless syringe. After infiltration, plants were stored at 24 °C for 50 h before GFP detection. To stain the nuclei, 10 μg/mL 4′,6-diamidino-2-phenylindole (DAPI) was infiltrated into *N. benthamiana* leaves 2 h before the observation point.

### Induction of anthocyanins by transient transformation of tobacco

Two-week-old seedlings of *N. benthamiana* grown in a greenhouse were used for infiltration. *Agrobacterium* strain GV3101 was selected for the transient assay. Separate strains containing *CsAN1, CsGL3, CsEGL3* and *CsTTG1* fused to the 35S promoter were cloned into the pBI121 vector. Infiltrations comprising the indicated constructs were infiltrated into the abaxial leaf surface. Each infiltration was performed using three leaves on the same plants, and at least ten plants were used for the analysis. For expression analysis, RNA was isolated from the *N. benthamiana* leaves at 4 days after infiltration. The anthocyanin content was measured after 10 days as described above.

### Isolation and identification of the *CsAN1* gene promoters

The promoter region of *CsAN1* was isolated using hiTAIL-PCR as described previously^47^. The analysis of the promoter *cis*-elements was performed using the online software PlantCARE (bioinformatics.psb.ugent.be/webtools/plantcare) and Place (www.dna.affrc.go.jp/PLACE/index).

### Transactivation assay of CsAN1

The full length *CsAN1* ORF and a series of truncated ORF fragments were amplified using specific primers that flanked both termini of each full length/truncated sequence (Table S1), and these amplified sequences were inserted into the pGBKT7 vector (Clontech). Each of the positive plasmids and an insert-less vector were transformed separately into the AH109 yeast strain (Clontech), according to the supplier’s protocol. Yeast colonies growing in SD/-Trp medium were transferred to SD/-Trp/-His/-Ade/X-α-gal medium after 3 d of culture at 30 °C, and the transactivation activity of each protein was evaluated according to their growth status and the activity of α-galactosidase.

### Yeast one-hybrid (Y1H) assay

Y1H assays were performed using the Matchmaker Gold Yeast One-Hybrid System Kit (Clontech) according to the manufacturer’s protocols. The *CsAN1* gene was ligated to pGADT7 to generate the AD-CsAN1 construct. Fragments of the *CsLDOX1*and *CsLDOX2* promoters were ligated to the pAbAi vector to generate pAbAi-bait plasmids, which were then linearized, transformed into the AH109 yeast strain, and selected with a selective synthetic dextrose medium lacking uracil. The AD-CsAN1 constructs were transformed into the AH109 strain containing pAbAi-bait and screened on an SD/-Leu plate containing 250 ng/ml Aureobasidin A (AbA).

### Dual luciferase assay of transiently transformed *N. benthamiana* leaves

A fragment containing 812 bp upstream of the ATG of the CsLDOX2 gene (AB074485) was isolated and inserted into the cloning site of pGreenII 0800-LUC. Transient transformation of the promoter-LUC fusion and luminescence measurements were performed as described previously^11^.

### Yeast two-hybrid (Y2H) assay

For the Y2H assay, all the CDS of *CsAN1, CsGL3, CsEGL3, CsTTG1* and their domain derivatives were cloned into pGADT7 or pGBKT7 vectors. Primers used for the vector construction are presented in Table S1. The Gold Yeast Two-Hybrid System was used for Y2H, according to the manufacturer’s protocols (Clontech). Y2H images were taken 4 days after incubation at 30 °C.

### Bimolecular fluorescence complementation (BiFC) assays

For BiFC assays, full-length coding sequences of *CsAN1, CsGL3, CsEGL3* and *CsTTG1* were cloned into the binary pSPYNE or pSPYCE vector using a ClonExpress II One Step Cloning Kit according to the manufacturer’s protocols (Vazyme). Primers used to generate the constructs are listed in Table S1. The GV3101 *Agrobacterium* strains with the indicated pSPYNE or pSPYCE vectors were incubated, harvested, and resuspended in infiltration buffer (0.2 mM acetosyringone, 10 mM MgCl_2_, and 10 mM MES). Equal concentrations and volumes of *Agrobacterium* strains were mixed and co-infiltrated into *N. benthamiana* leaves using a needleless syringe. After infiltration, the plants were placed at 24 °C for 50 h before observation. The experiments were repeated three times.

### Environmental conditions

Temperature treatments were conducted on three-year-old ‘ZJ’ plants, which were grown in a plant growth chamber at 15–28 °C with a 12 h light/12 h dark period and a relative air humidity of 60%. The leaves were harvested 15 days after budding (DAB). For low temperature-induced anthocyanin biosynthesis, three-year-old ‘ZJ’ tea plants were grown in a plant growth chamber under the following conditions: 15 °C and a 12 h photoperiod. All samples were collected at the indicated times after treatment, frozen in liquid nitrogen and stored at −80 °C.

For light treatment, three-year-old ‘ZJ’ trees were grown at 18 °C for 6 h, 12 h, 18 h and 24 h light periods. The leaves were harvested after 15 DAB, frozen in liquid nitrogen and stored at −80 °C.

### 5-aza-dC treatment

*C. sinensis* plants were sprayed with 20 mM Tris-HCl, pH 7.5, with or without 5 mM 5-aza-dC (Sigma), and were maintained at room temperature for 48 h in the dark. After treatment for 7 days, stage S2 leaves were sampled for further analysis.

### Methylation analysis

BSP-PCR analysis was performed as described previously^48^. Briefly, 500 ng of genomic DNA from S2 stage leaves was treated with the EZ DNA Methylation-Gold Kit (Zymo Research). Using the treated DNA as a template, CsAN1 promoter fragments were amplified using PrimeSTAR Max DNA polymerase (TaKaRa), ligated to the PMD19-T vector (TaKaRa), and then sequenced. Sequences of 15 independent clones of the target fragment were obtained from three independent PCR reactions and analyzed with the online software Kismeth^31^. The methylation level of each fragment was calculated. To further examine whether the methylation levels are dependent on the leaf development stage, bisulfite sequencing analysis was also performed using *g*DNA from ‘ZJ’ leaf samples at the four stages as above. Three independent PCR reactions from three different biological replicates were purified and sequenced for analysis.

To further detect whether environmental factors influence CsAN1 promoter methylation, three-year-old ‘ZJ’ tea plants were grown in a plant growth chamber at 15 °C, 18 °C, 23 °C and 28 °C with a 12 h light/12 h dark period and relative air humidity of 60%. The S2 stage leaves were harvested for methylation analysis as described above. To examine whether the methylation was influenced by the photoperiod, three-year-old ‘ZJ’ trees were grown at 18 °C for 6 h, 12 h, 18 h and 24 h light periods. The S2 stage leaves were harvested for further methylation analysis as described above.

## Additional Information

**How to cite this article**: Sun, B. *et al*. Purple foliage coloration in tea (*Camellia sinensis* L.) arises from activation of the R2R3-MYB transcription factor CsAN1. *Sci. Rep.* **6**, 32534; doi: 10.1038/srep32534 (2016).

## Supplementary Material

Supplementary Information
