# Supplementary material for: Purple foliage coloration in tea (Camellia sinensis L.) arises from activation of the R2R3-MYB transcription factor CsAN1
Source: Sci Rep. 2016 Sep 1;6:32534. doi: 10.1038/srep32534 (PMC5007479; doi:10.1038/srep32534)
Supplement: Supplementary Information [file srep32534-s1.doc]

**Purple foliage** **coloration in** **tea (*****Camellia sinensis* L.) arises from activation of** **the R2R3-MYB transcription factor CsAN1**

Binmei Sun^1^, *, Zhangsheng Zhu^1^, *, Panrong Cao^1^, *, Hao Chen^1^, Changming Chen^1^, Xin Zhou^1^, Yanhui Mao^1^, JianJun Lei^1^, Yanpin Jiang^1^, Wei Meng^2^, Yingxi Wang^1^, Shaoqun Liu^1^

^1^ College of Horticulture, South China Agricultural University, Guangzhou 510642, China

^2^ College of Forestry and Landscape Architecture, South China Agricultural University, Guangzhou 510642, China

* These authors contributed equally to this work.

Correspondence and requests for materials should be addressed to Shaoqun Liu (Email: scau@163.com)

**Supplementary Information**


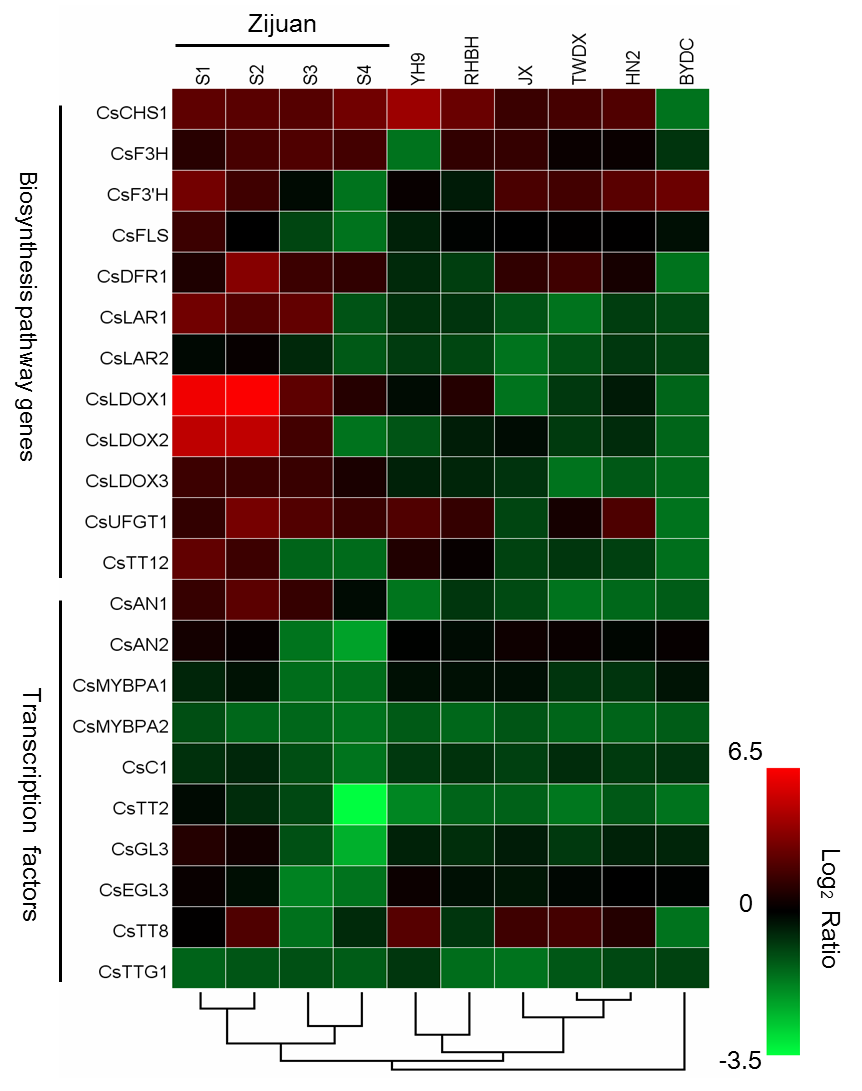


**Supplementary** **Fig. S1** Expression profiles of *C. sinensis* anthocyanin biosynthetic genes and putative transcription factors. Real-time PCR was used to analyse genes expression patterns in leaves of the *Camellia sinensis* cultivars ‘Zijuan’ (ZJ), ‘Yinghong9’ (YH9), ‘Renhuabaihao’ (RHBH), ‘Jinxuan’ (JX), ‘Taiwandanxuan’ (TWDX), ‘Huanong’ (HN2), ‘Baiyedancong’ (BYDC). The *Actin* was used as the reference gene. S1, bud; S2, 7 days after budding (DAB); S3, 14 DBA; S4, 40 DAB.


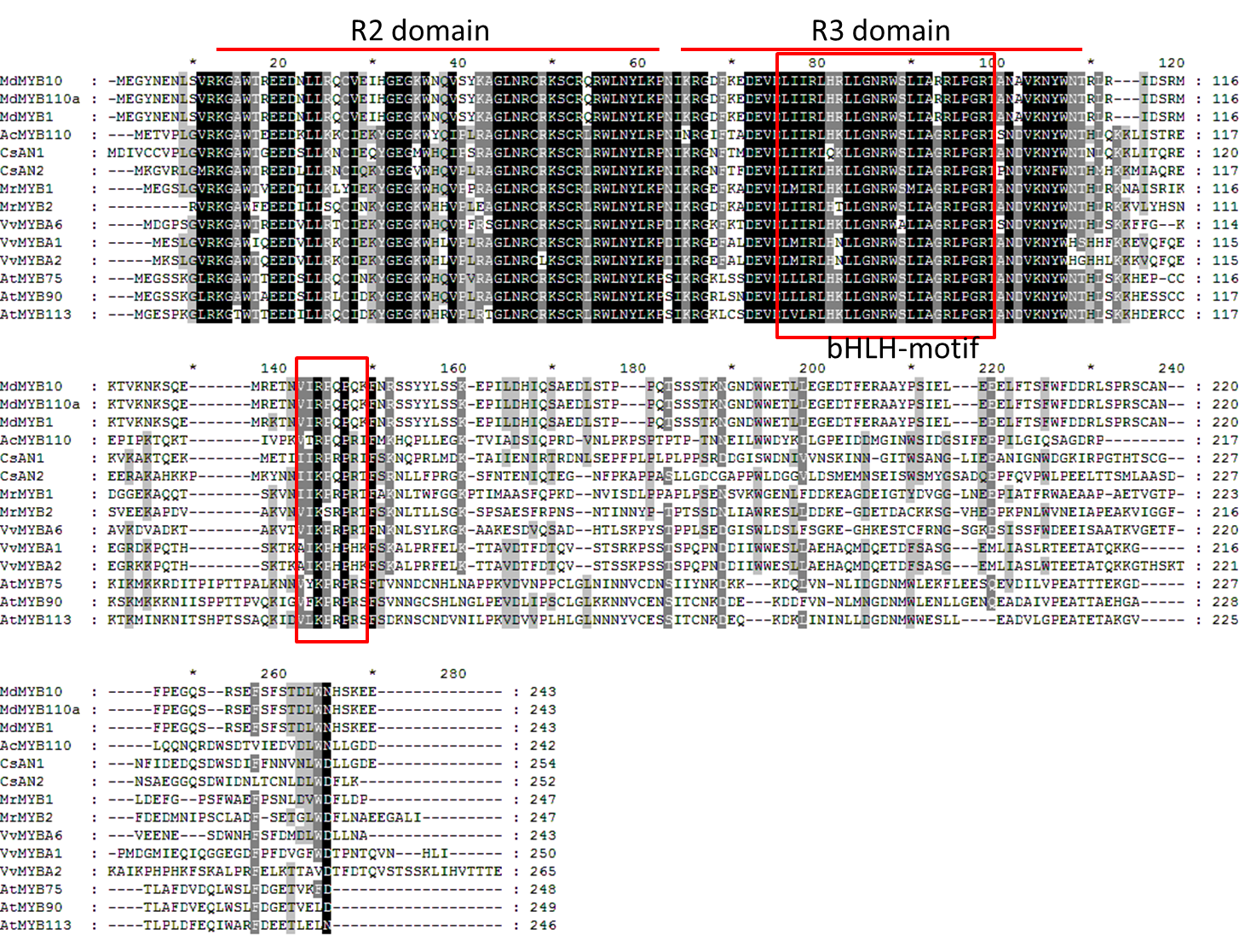


**Supplementary Fig. S2** Proteins sequence alignment of the CsAN1, CsAN2 and the other known anthocyanin related R2R3-MYB regulators in other species.


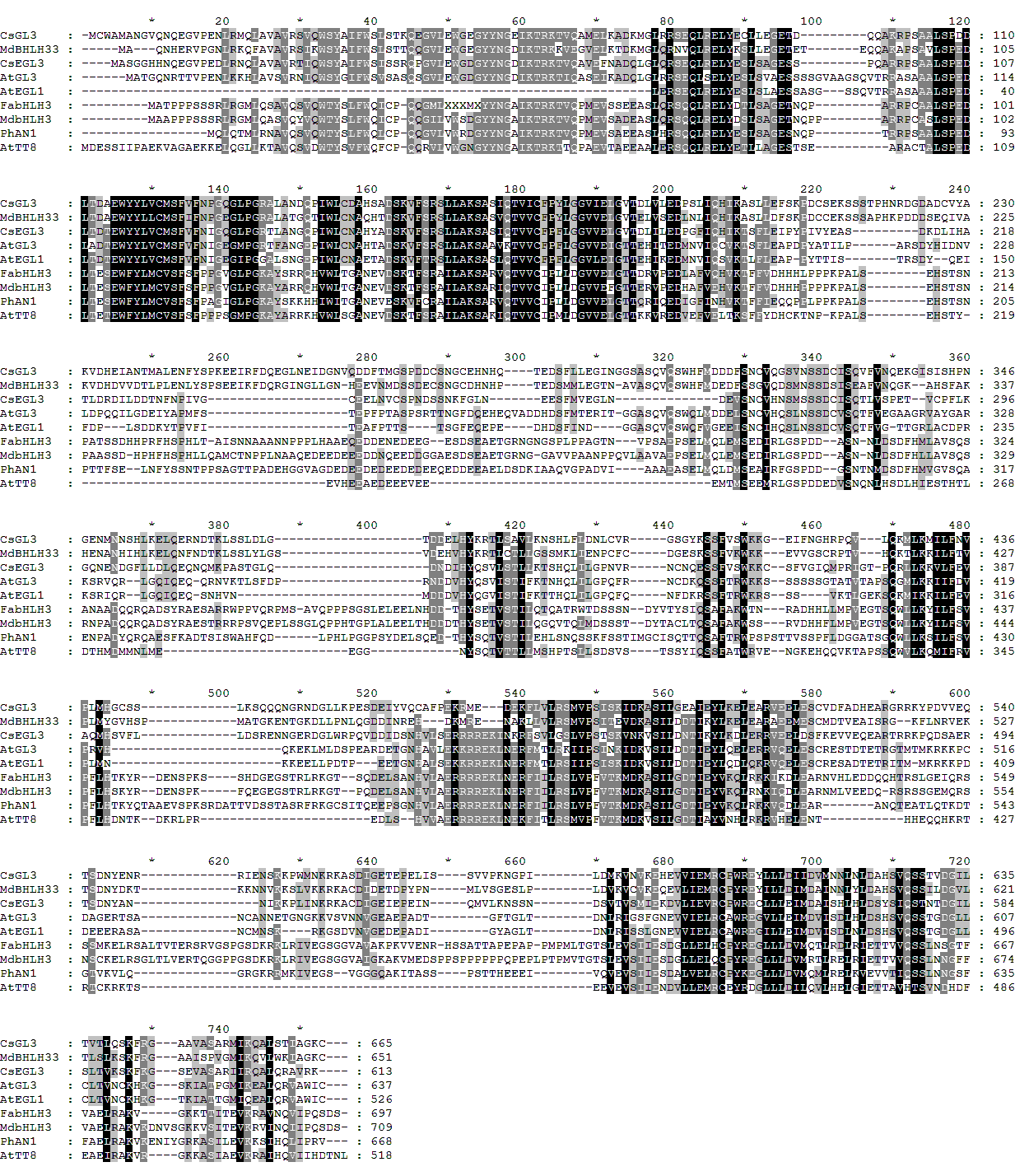


**Supplementary Fig. S3** Proteins sequence alignment of the CsGL3, CsEGL3 and the other known anthocyanin related bHLH regulators in other species.


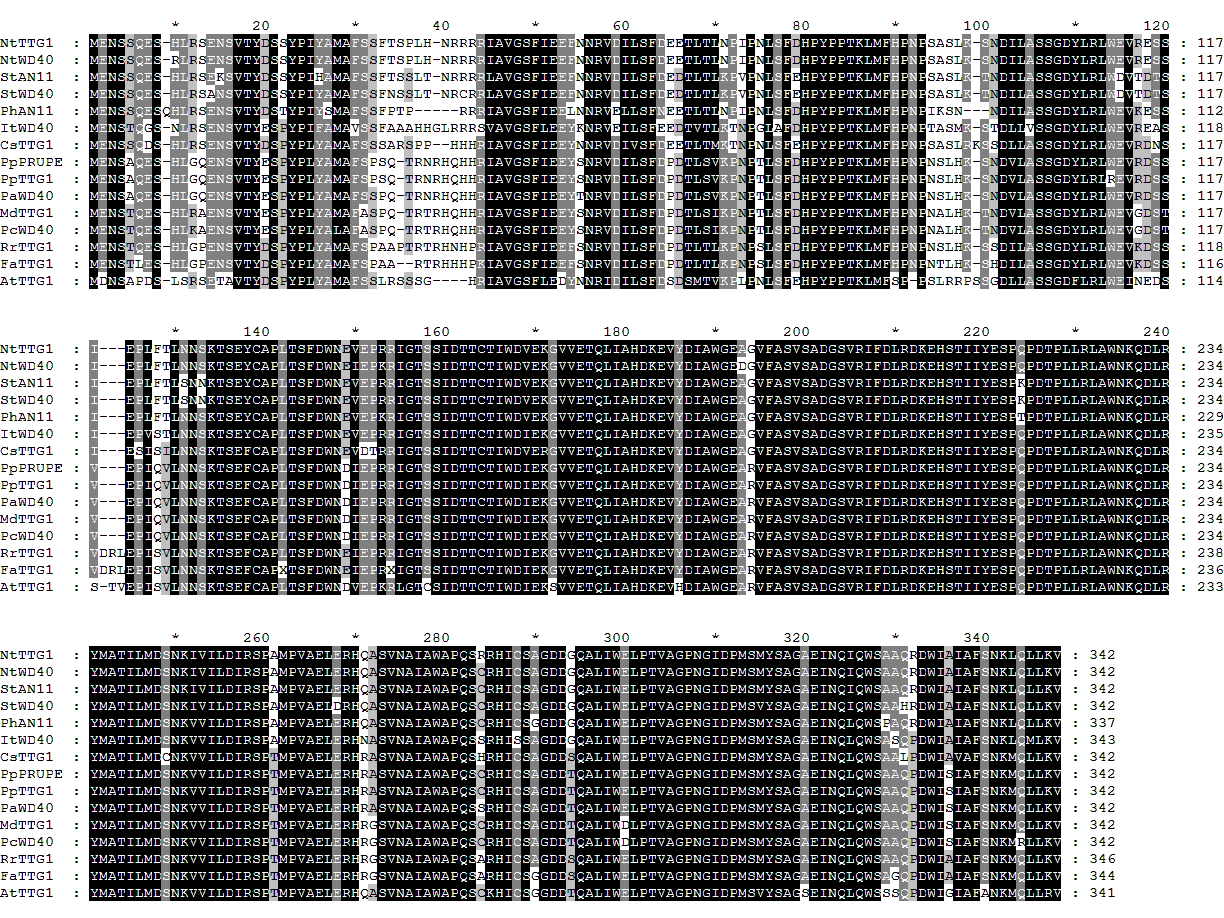


**Supplementary Fig. S4**  Protein sequence alignment of the CsTTG1 and the TTG1-like protein in other species.


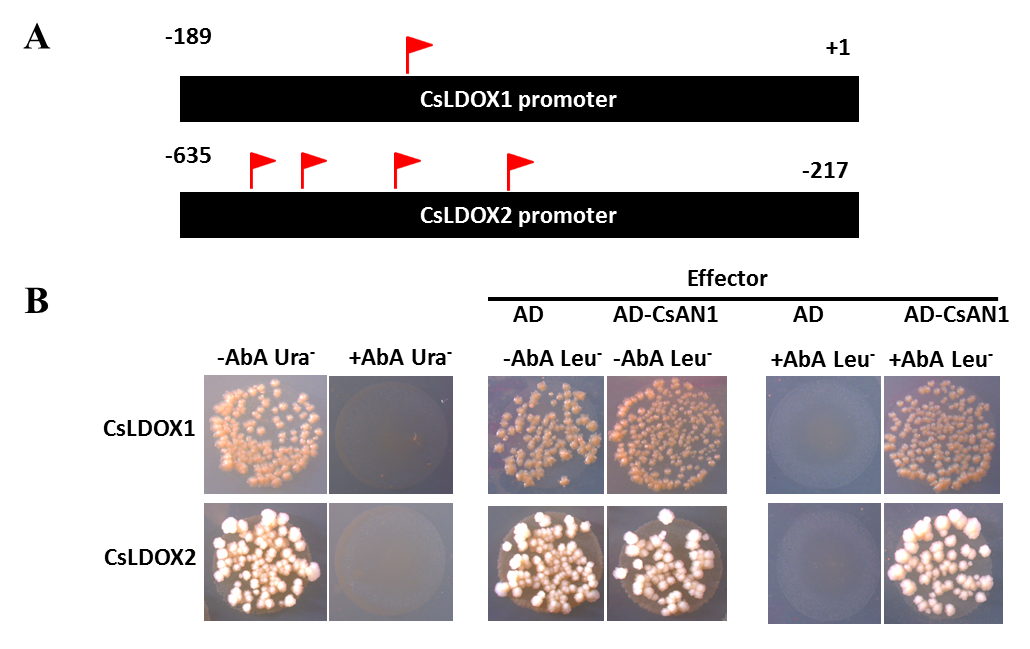


**Supplementary Fig. S5** The association of CsAN1 with the promoters of CsLDOX1 and CaLDOX2 by Yeast one-hybrid (Y1H) assay. (**A**) Diagrams of the CsLDOX1 and CaLDOX2 promoter for Y1H. Red flag, MYB-banding site (MBS). (**B**) No basal activities of CsLDOX1 and CsLDOX2 promoters was detected in yeast grown on SD medium lacking Leu in the presence of AbA. Yeast growth assays after the yeast Y1H reporter strains harboring the promoters were transformed with plasmids carrying cassettes constitutively expressing CaAN1 effector or empty (AD, negative control). Interaction was determined based on the ability of transformed yeast to grow on SD medium lacking Leu in the presence of Aureobasidin A (AbA)


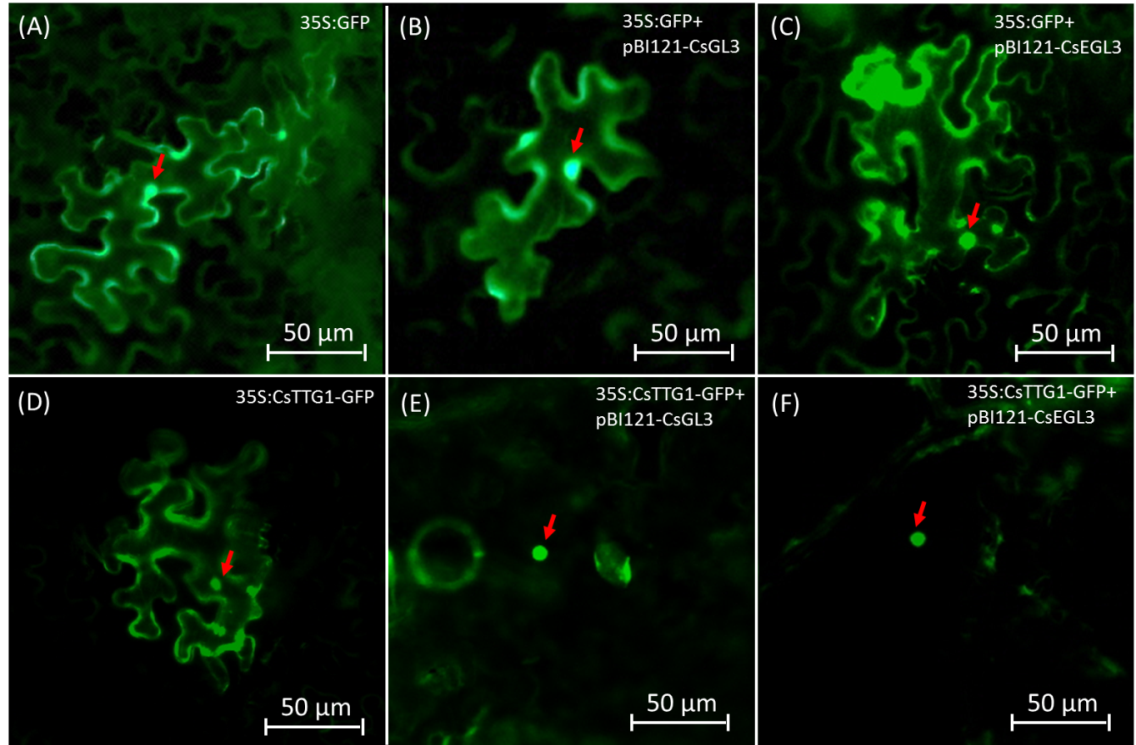


**Supplementary Fig. S6** Cellular distribution pattern of CsTTG1-GFP fusion protein in the *N. benthamiana* leaf epidermis. (A) 35S:GFP. (B) and (C), Co-expression of 35S:GFP with 35S:CsGL3 or 35S:CsEGL3. (D) 35S:CsTTG1-GFP. The GFP fluorescence intensity is much weaker in the nuclei of leaf epidermis cells. (E) Co-expression of CsTTG1-GFP and 35S:CsGL3. (F) Co-expression of CsTTG1-GFP and 35S:CsEGL3. More than 20 cells in six independent experiments exhibited the intracellular localisation patterns shown here.


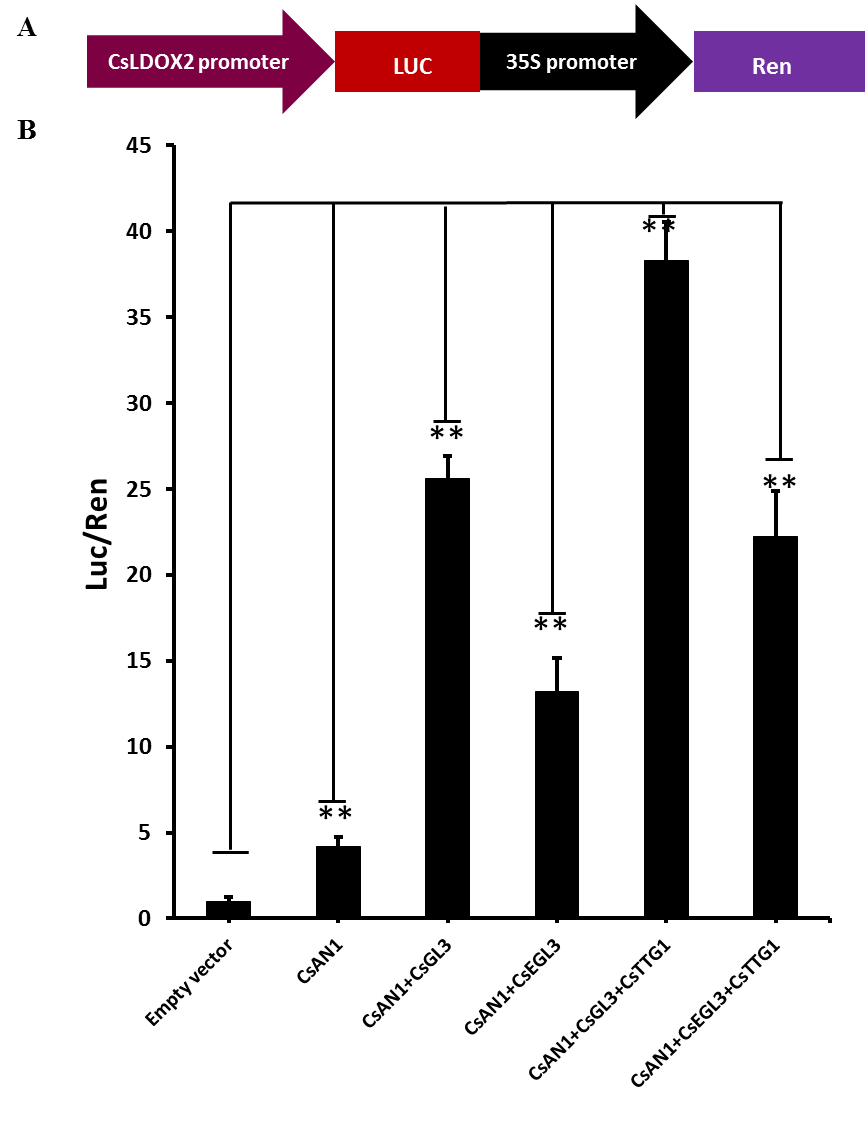


**Supplementary Fig. S7** Transient assays demonstrate the function of CsAN1 as a regulator of anthocyanin biosynthesis. (**A**) Schematic diagram of *CsLDOX2* promoter construct. (**B**) Leaves of *N. benthamiana* were infiltrated with the *CsLDOX2* promoter-LUC fusions on their own or co-infiltrated with 35S:CsAN1, with or without 35S:CsGL3 and 35S:CsEGL3 and 35S:CsTTG1. Luminescence of luciferase and renilla was measured 3 d later and expressed as a ratio of luciferase to renilla. Data are presented as means of four replicate reactions. Asterisks indicate significant difference (P < 0.01) between combinations by Student’s t-test.


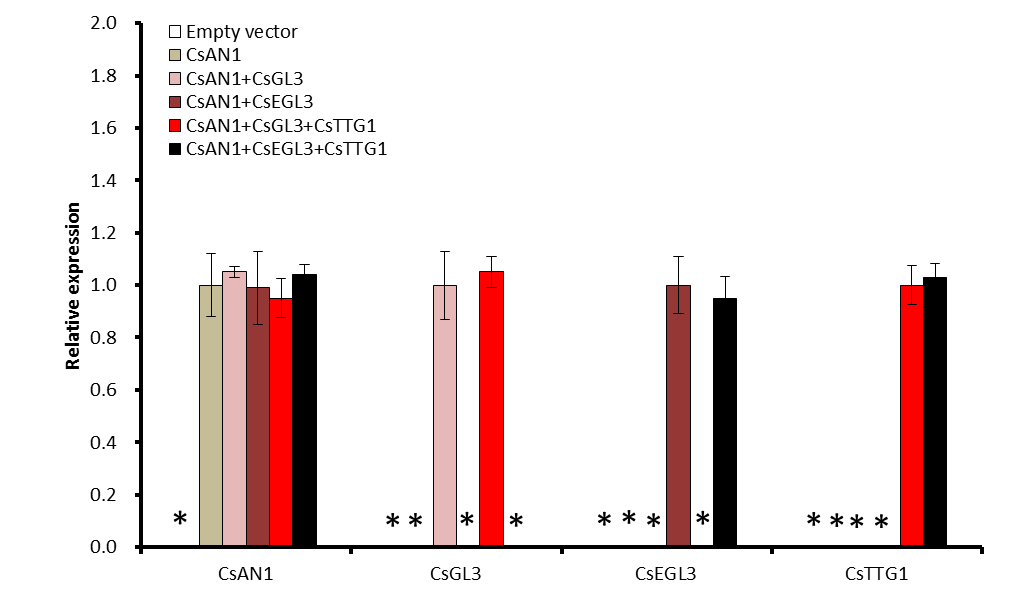


**Supplementary Fig. S8** Transient overexpression of *C.sinensis* MBW complex genes induced anthocyanin biosynthesis in *N. benthamiana* leaves. qRT-PCR analysis of *C.sinensis* MBW complex genes genes in *N. benthamiana* leaves transformed with indicated vectors (*CsAN1*, *CsAN1*+*CsGL3*, *CsAN1*+*CsEGL3*, *CsAN1*+*CsGL3*+*CsTTG1*, *CsAN1*+*CsEGL3*+*CsTTG1* and empty vector) in 4 days. The asterisks represent no detected gene expression. Error bars are the SD for five replicate reactions.


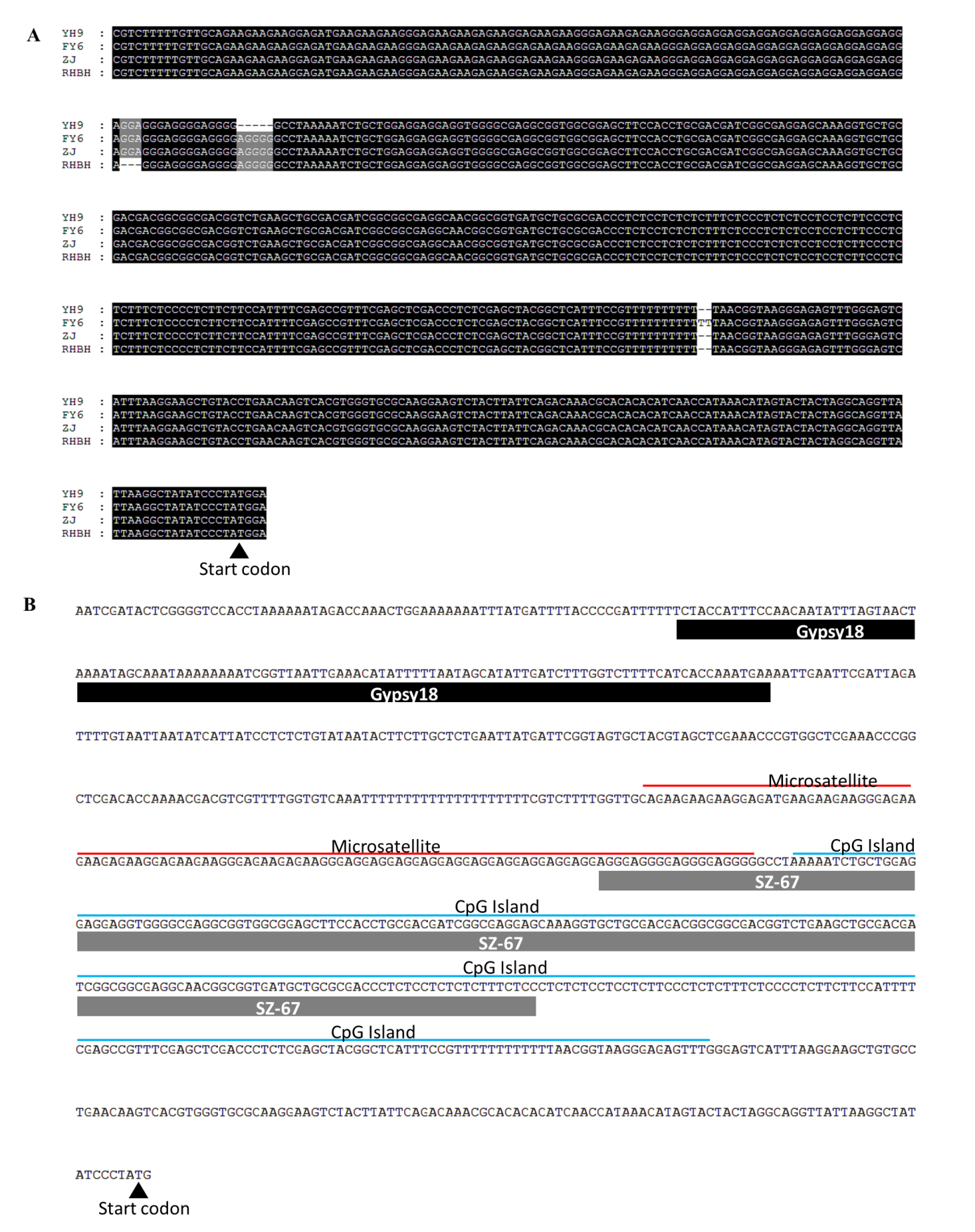


**Supplementary Figure S9.** Analysis of CsAN1 promoter. (**A**) Sequence alignment of CsAN1 promoters in four cultivars.YH9, ‘Yinghong 9’; FY6, ‘Fuyun 6’; ZJ, ‘Zijuan’, RHBH, ‘Renhuabaihao’. (**B**) Analysis of ‘Zijuan’ tea CsAN1 promoter elements.


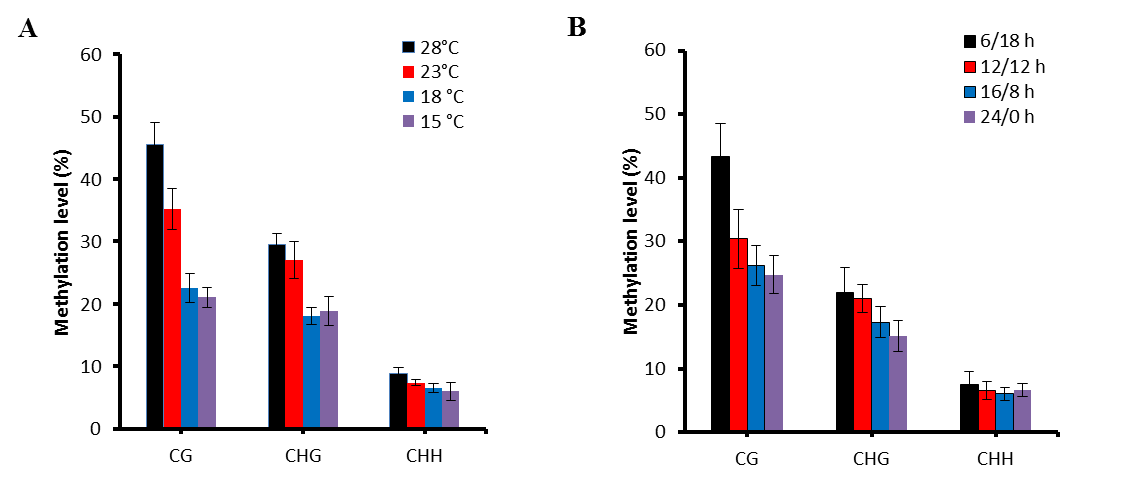


**Supplementary** **Figure S10.** DNA methylation analysis of the ‘Zijuan’ tea CsAN1 promoter under differential environmental conditions. (**A**) The stage S2 leaves DNA methylation status of CpG island treatment in 15 °C, 18°C, 23°C and 28°C, respectively. (**B**) The stage S2 leaves DNA methylation status of CpG island under different photoperiod conditions.

**Supplementary Table S1.** List of oligonucleotide primers and sequences

| Primer名称 | Sequence (5’-3’) | Description |
| --- | --- | --- |
| CsCHS1-F | ACATACACACCACCACACCTA | qPCR |
| CsCHS1-R | TTGCCATTCCAATCGCCATAA | qPCR |
| CsFL3H-F | CCTTCCAATCCCCCTTCCTTCA | qPCR |
| CsFL3H-R | TGTCCTTGCTCTCCCAACACCT | qPCR |
| CsF3'H-F | AATAACCATTCACTCGGCAAGC | qPCR |
| CsF3'H-R | CACGACCAACATCAAGGCACTA | qPCR |
| CsFLS-F | GGGACAAGTAAAGTGAGAGCA | qPCR |
| CsFLS-R | TTCATACAGGGGAGTGACAGA | qPCR |
| CsDFR1-F | ACTGGATGGATGTATTTTGTGG | qPCR |
| CsDFR1-R | TAGAGGGTTGAATGGTGATGAG | qPCR |
| CsLAR1-F | GGTTGATAGAGGAGTCAGGGGT | qPCR |
| CsLAR1-R | AATTGCAAGATGGTCGGAAATG | qPCR |
| CsLAR2-F | CGGCGATTGATAGAAGAGTGT | qPCR |
| CsLAR2-R | GAACCGATTTGTTCAGCGTAC | qPCR |
| CsLDOX1-F | GCTCCTCCTCCGTCACCGTCT | qPCR |
| CsLDOX1-R | CGTCCCCAACTCCATTATCAT | qPCR |
| CsLDOX2-F | GCGGTGGATTGGGGTGTGATG | qPCR |
| CsLDOX2-R | TAGTCGGTTGGTGTCTTGGGC | qPCR |
| CsLDOX3-F | TGGTGCTTGTTTGAGGGTGAA | qPCR |
| CsLDOX3-R | GCATTAGGGGCAGGCTTGACT | qPCR |
| CsUFGT1-F | TGTCTTCTCTATTGAGTCTGC | qPCR |
| CsUFGT1-R | GAAAGTTTAAAGTTTGGGGT | qPCR |
| CsTT12-F | TGGCTGAAGAAACTACCAAAC | qPCR |
| CsTT12-R | ACTAAGTAATAGCAACCGATG | qPCR |
| CsAN1-F | TCTTCTCTTGAGTCTTGGCT | qPCR |
| CsAN1-R | ATAGATGGTCGTTGATTGCG | qPCR |
| CsAN2-F | AAGCTGTAGATTGAGGTGGTT | qPCR |
| CsAN2-R | GGGAAGTCTGCCTGCTATTAG | qPCR |
| CsC1-F | AGAGAGGAAACATTAGCCGTG | qPCR |
| CsC1-R | TTGGATTGAGGATTTGAGGAG | qPCR |
| CsMYBPA1-F | CTGGTTTGAAGCGATGTGGTAA | qPCR |
| Cs MYBPA1-R | TGCGGTCGGACTCTCTGGTGAT | qPCR |
| Cs MYBPA2-F | CAAATGGACTTAGAAAGGGGTG | qPCR |
| Cs MYBPA2-R | GCTTGATGTTCGGTCTGAGATA | qPCR |
| CsTT2-F | TTCATGCAACTCACTACTC | qPCR |
| CsTT2-R | TAAACTCCTCACAAACCTT | qPCR |
| CsGL3-F | AGAAGGGTTGAGGAGTTGGA | qPCR |
| CsGL3-R | TGTTGTTGGCGTAGTTATCG | qPCR |
| CsEGL3-F | CTTTTGAAACCAGAAAGTG | qPCR |
| CsEGL3-R | TGCTAATAGAAGGAACCAT | qPCR |
| CsTT8-F | TTTGCCTGTTTCCATCTTAG | qPCR |
| CsTT8-R | GATTTTCATCGCATCTTGTG | qPCR |
| CsTTG1-F | AACTCCATCGAATCCATCTC | qPCR |
| CsTTG1-R | AACCTCTTTATCGTGGGCAA | qPCR |
| CsActin-F | GCCATATTTGATTGGAATGG | qPCR |
| CsActin-R | GGTGCCACAACCTTGATCTT | qPCR |
| CsTubulin -F | CACATTTGTTGGGTTAGTTCGG | qPCR |
| CsTubulin-R | CATTCCCTCGTCTCCATTTCTT | qPCR |
| NbF3’H-F | TAAGGCTTCATCCATCCACC | qPCR |
| NbF3’H-R | CAAAGTCATTTCCTCGCACA | qPCR |
| NbDFR-F | TTGTTGGTCCATTCCTCACG | qPCR |
| NbDFR-R | CCACGGGCAAGTCCTTATCG | qPCR |
| NbLDOX-F | AGTATGCTAATGACCAACCCTC | qPCR |
| NbLDOX-R | AGTCCCAGCCCAATAGAAAG | qPCR |
| NbActin-F | AGTCCTCTTCCAGCCATCCA | qPCR |
| NbActin-R | TAGGAGCCAAAGCCGTGATT | qPCR |
| pBI121-CsAN1-F | GGACTCTAGAGGATCCATGGACATTGTTTGTTGTGT | Overexpression |
| pBI121-CsAN1-R | GACCACCCGGGGATCCTCATCATTCATCACCTAACA | Overexpression |
| pBI121-CsEGL3-F | GGACTCTAGAGGATCCATGTGTTGGGCAATGGCT | Overexpression |
| pBI121-CsEGL3-R | GACCACCCGGGGATCCCTAACACTTGCCAGCAAT | Overexpression |
| pBI121-CsGL3-F | GGACTCTAGAGGATCCATGGCTTCTGGGGGGCACC | Overexpression |
| pBI121-CsGL3-R | GACCACCCGGGGATCCTCACTTTCTAACCGCTCTCT | Overexpression |
| pBI121-CsTTG1-F | GGACTCTAGAGGATCCATGGAGAATTCGAGCCAAG | Overexpression |
| pBI121-CsTTG1-R | GACCACCCGGGGATCCTCAAACTTTCAGAAGCTGCA | Overexpression |
| PSPYNECsAN1-F | CGCCACTAGTGGATCCATGGACATTGTTTGTT | BiFC assay |
| pSPYNECsAN1-R | TACTATCGATGGATCCTCATTCATCACCTAACA | BiFC assay |
| pSPYCECsEGL3-F | CGCCACTAGTGGATCCATGTGTTGGGCAATGGCT | BiFC assay |
| pSPYCECsEGL3-R | TACTATCGATGGATCCCTAACACTTGCCAGCAAT | BiFC assay |
| pSPYCECsGL3-F | CGCCACTAGTGGATCCATGGCTTCTGGGGGGCACC | BiFC assay |
| pSPYCECsGL3-R | TACTATCGATGGATCCTCACTTTCTAACCGCTCTCT | BiFC assay |
| pSPYNECsTTG1-F | CGCCACTAGTGGATCCATGGAGAATTCGAGCCAAG | BiFC assay |
| pSPYNECsTTG1-R | TACTATCGATGGATCCTCAAACTTTCAGAAGCTGCA | BiFC assay |
| pEGFP- CsAN1-F | CGCGGGCCCGGGATCCATGGACATTGTTTGTT | Subcellular localization |
| pEGFP - CsAN1-R | GGCGACCGGTGGATCCTCATTCATCACCTAACA | Subcellular localization |
| pEGFP -CsEGL3-F | CGCGGGCCCGGGATCCATGTGTTGGGCAATGGC | Subcellular localization |
| pEGFP -CsEGL3-R | GGCGACCGGTGGATCCACACTTGCCAGCAAT | Subcellular localization |
| pEGFP -CsGL3-F | CGCGGGCCCGGGATCCATGGCTTCTGGGGGGCA | Subcellular localization |
| pEGFP -CsGL3-R | GGCGACCGGTGGATCCCTTTCTAACCGCTCTCT | Subcellular localization |
| pEGFP -CsTTG1-F | CGCGGGCCCGGGATCCATGGAGAATTCGAGCC | Subcellular localization |
| pEGFP -CsTTG1-R | GGCGACCGGTGGATCCAACTTTCAGAAGCTGCA | Subcellular localization |
| ADCsAN1-F | GGAGGCCAGTGAATTCATGGACATTGTTTGTTGTG | Yeast-tow- hybridization |
| ADCsAN1-R | CGAGCTCGATGGATCCTCATTCATCACCTAACAGA | Yeast-tow- hybridization |
| ADCsAN1NT-R | CGAGCTCGATGGATCCCATTAACCGTGGTTGATTT | Yeast-tow- hybridization |
| ADCsAN.1CT-F | GGAGGCCAGTGAATTCATACGACCTCGGCCTCGA | Yeast-tow- hybridization |
| BDCsGL3-F | CATGGAGGCCGAATTCATGGCTTCTGGGGGGCAC | Yeast-tow- hybridization |
| BDCsGL3-R | GCAGGTCGACGGATCCTCACTTTCTAACCGCTCTC | Yeast-tow- hybridization |
| BDCsGL3NT-R | GCAGGTCGACGGATCCCTAGCCCAAACTTGTTTGA | Yeast-tow- hybridization |
| BDCsGL3CT-F | CATGGAGGCCGAATTCGATCGGGACATTCTTGATG | Yeast-tow- hybridization |
| BDCsEGL3-F | CATGGAGGCCGAATTCATGTGTTGGGCAATGGCTA | Yeast-tow- hybridization |
| BDCsEGL3-R | GCAGGTCGACGGATCCCTAACACTTGCCAGCAATAG | Yeast-tow- hybridization |
| BDCsEGL3NT-R | GCAGGTCGACGGATCCTCAGATTTCTTCTTTCGGGGA | Yeast-tow- hybridization |
| BDCsEGL3CT-F | CATGGAGGCCGAATTCTCTACTCCTCATAACAGAGA | Yeast-tow- hybridization |
| ADCsTTG1-F | GGAGGCCAGTGAATTCATGGAGAATTCGAGCCAAG | Yeast-tow- hybridization |
| ADCsTTG1-R | CGAGCTCGATGGATCCTCAAACTTTCAGAAGCTGC | Yeast-tow- hybridization |
| BDCsTTG1-F | CATGGAGGCCGAATTCATGTGTTGGGCAATGGCTA | Yeast-tow- hybridization |
| BDCsTTG1-R | GCAGGTCGACGGATCCTCAAACTTTCAGAAGCTGC | Yeast-tow- hybridization |
| pAbAi-LDOX1-F | TTGAATTCGAGCTCGGTACC TTCCTTCTTATTTGGTAGCT | Yeast-one- hybridization |
| pAbAi-LDOX1-R | TCGACAGATCCCCGGGTACC CCATTTTTTCAAACTCAAT | Yeast-one- hybridization |
| pAbAi-LDOX2-F | TTGAATTCGAGCTCGGTACCCGGGCTGGTATCTTTCATATTC | Yeast-one- hybridization |
| pAbAi-LDOX2-R | TCGACAGATCCCCGGGTACCCTTTGGTAAAGGTAATGAATG | Yeast-one- hybridization |
| BDCsAN1-F | CGGAATTCATGGACATTGTTTGTTGTG | Transactivation |
| BDCsAN1-R | CGGGATCCTCATTCATCACCTAACAGA | Transactivation |
| BDCsAN1-10-F | CGGAATTCGTGAGAAAAGGTGCATGGA | Transactivation |
| BDCsAN1-67-F | CGGAATTCAATTTTACGATGGATGA | Transactivation |
| BDCsAN1-127-F | CGGAATTCGAGAAGATGGAAACCAT | Transactivation |
| BDCsAN1-195-F | CGGAATTCAATGGAATCACATGGTC | Transactivation |
| BDCsAN1-194-R | CGGGATCCGTTAATTTTGGAGTTAAC | Transactivation |
| pGreen800LUC-LDOX2-F | TATAGGGCGAATTGGCATATTCATATATTTTCTGAACATA | Dual luciferase assay |
| pGreen800LUC-LDOX2-R | TAGAACTAGTGGATCGTCATCATAACCATGTAGTACACCA | Dual luciferase assay |
| BSP-CsAN1-F | GTTTAAAAATTTGTTGGAGGAGGA | BSP-PCR |
| BSP- CsAN1-R | AACACAACTTCCTTAAATAACTCCC | BSP-PCR |

**Supplementary Table S2.** Analysis of CsAN1 promoter*cis*-elements

| Motif Name | Location in the Promoter | Sequence | Function |
| --- | --- | --- | --- |
| MYBCORE | -145,-289, -78 | CNGTTR | MYB banding site |
| MYBCOREATCYCB1 | -160,-198 | AACGG | MYB banding site |
| MYBST1 | -3,-684 | GGATA | MYB banding site |
| E-box/G-box | -92, -369, -726 | CANNTG | MYC recognition site |
| ABRELATERD1 | -93 | ACGTG | Dehydration stress and dark-induced senescence |
| WRKY71OS | -96, -124 | TGACT | WRKY proteins bind site |
| GT1CONSENSUS | -206,-552,-685, -779 | GRWAAW | light-regulated elemnts |
| GCCCORE | -299, -328 | GCCGCC | ethylene-responsive element |
| WBOXATNPR1 | -574 | TTGAC | Salicylic acid (SA)-induced WRKY binding site |
| CGTCA-motif | -600 | CGTCA | MeJA-responsiveness |
| IBOXCORE | -685 | GATAA | Conserved sequence upstream of light-regulated genes ofboth monocots and dicots |
| AuxRE | -64, -586, -592 | TGTCTCAATAAG | Auxin-responsive element |
| ABRE | -94, | ACGTGGC | Abscisic acid responsiveness |
| Sp1 | -430, -435, -440, -474 | CC(G/A)CCC | Light responsive element |
| TCA-element | -504 | GAGAAGAATA | Salicylic acid responsiveness |
| G-rich box | 129, 139, 225, 243, 246, -254, 267, -354, -381, -403, -406, -409, -431, -436, -441, -445, -448, -451, -454, -457, -460, -463, -466, -469, -472, -475, -487, -497, -511, -527 | GGAG | Methylation protection |
